# Protein disorder reduced in *Saccharomyces cerevisiae *to survive heat shock

**DOI:** 10.12688/f1000research.7178.1

**Published:** 2015-11-06

**Authors:** Esmeralda Vicedo, Zofia Gasik, Yu-An Dong, Tatyana Goldberg, Burkhard Rost

**Affiliations:** 1 Department of Informatics, Bioinformatics & Computational Biology, TUM, Munich, Germany; 2 Graduate School of Information Science in Health, TUM, Munich, Germany; 3 Institute of Experimental Physics, Division of Biophysics, University of Warsaw, Warsaw, Poland; 4 Institute of Systems Biology, Shanghai University, Shanghai, China; 5 Institute of Advanced Study, TUM, Munich, Germany; 6 Institute for Food and Plant Sciences WZW, TUM, Freising, Germany

**Keywords:** Saccharomyces cerevisiae, heat-shock stress, chromosomal duplication, GO terms, protein disorder, protein-protein interactions, heat shock proteins

## Abstract

Recent experiments established that a culture of *Saccharomyces cerevisiae* (baker’s yeast) survives sudden high temperatures by specifically duplicating the entire chromosome III and two chromosomal fragments (from IV and XII). Heat shock proteins (HSPs) are not significantly over-abundant in the duplication. In contrast, we suggest a simple algorithm to “ *postdict* *”* the experimental results: Find a small enough chromosome with minimal protein disorder and duplicate this region. This algorithm largely explains all observed duplications. In particular, all regions duplicated in the experiment reduced the overall content of protein disorder. The differential analysis of the functional makeup of the duplication remained inconclusive. Gene Ontology (GO) enrichment suggested over-representation in processes related to reproduction and nutrient uptake. Analyzing the protein-protein interaction network (PPI) revealed that few network-central proteins were duplicated. The predictive hypothesis hinges upon the concept of reducing proteins with long regions of disorder in order to become less sensitive to heat shock attack.

## Introduction

*Saccharomyces cerevisiae* (baker’s yeast; for simplicity we mostly use yeast) was the first completely sequenced eukaryote ^1^. Being simple to handle and manipulate has rendered yeast a preferred model organism for genetics, biochemistry and systems biology ^2– 4^. It grows optimally within a narrow temperature range but tolerates moderate deviations, some of which impinge upon cell structure and function, often through rapid physiological adaptations. One such adaptation mechanism is the duplication of the whole genome or particular chromosomes (aneuploidy) ^5– 7^ that contain the genes necessary to rapidly cope with specific adverse conditions over the course of several generations of evolving yeast ^8– 14^. Such evolutionary adaptations imbalance the genome ^15^, destabilize reactions or pathways ^16, 17^, and cost energy ^18, 19^. Aneuploidy, therefore, is a transient solution. Over many generations exposed to the same adverse conditions refined specific and less expensive solutions replace aneuploidy ^20^. Yeast cells can adapt to high-temperature stress by repeatedly duplicating chromosome III along with two other fragments (from chrIV and chrXII) ^20^. Why specifically copy these regions? Can particular biophysical features and/or functions of the proteins encoded in these regions explain the choice?

One simple biophysical feature is protein disorder: most proteins adopt well-defined three-dimensional (3D) structures ^21– 24^, *i.e.* will largely remain identical at different times. In contrast, *disordered regions* do not adopt well-defined 3D structures in isolation ^25^, *i.e*. without binding substrates they will look very different at different time points. Proteins with long disordered regions encompass some unique biophysical characteristics ^26– 34^. Such regions are so difficult to characterize experimentally that there is no good experimental data set proxy for “all proteins with long regions of disorder in yeast”. In contrast, acceptably accurate computational predictions are available for entire proteomes ^30, 35, 36^. Protein disorder seems one means for prokaryotes to adopt to extreme environments, e.g. halophiles have more proteins with long disorder than their closest phylogenetic relatives, while thermophiles tend to have fewer ^37^. Here, we hypothesized a similar effect to govern the response to high temperature-related duplication in yeast, namely that chromosomal regions duplicated under high temperature are depleted of proteins with long disorder.

## Methods

### Data

We downloaded the yeast ( *S. cerevisiae*) proteome from UniProt (proteome ID: UP000002311) ^38^ as fasta files including only the reviewed proteins (UniProtKB/Swiss-Prot). Removal of duplicates applying the method Uniqueprot2 ^39^ (with 100% pairwise sequence identity, keeping the longer sequence) left 5667 proteins ( Table S1A). We considered the 16 nuclear chromosomes (matched through http://www.yeastgenome.org ^40^, the numbers of proteins per chromosome are given in Figure S1B). The yeastgenome.org resource also provided the annotations of heat-shock response proteins (HSR). Proteins known to interact with HSR proteins augmented this set of HSRs in the following way.

BioGRID (version 3.1.86) provided the data for experimental protein-protein interactions (PPIs) in yeast. After filtering out redundancy (a-b and b-a counted only once) and excluding self-interactions (a-a), we based all subsequent analysis on the single largest connected component of the network. We focused on the most basic network features that allow the comparison and characterization of complex networks. The most elementary characteristic of a node is its degree or connectivity, defined as the number of interactions for a node (here protein), i.e. the number of interactions one protein has with all others. Another important parameter is the betweenness, *i.e*. the fraction of shortest paths between all other nodes that has to go through a given node. Additionally, we monitored the average degree of neighbors, which depends on the number of nodes and links in the network. These three parameters measured the importance of each node within the network.

### Disorder predictions

We applied methods capturing different “flavors” of protein disorder ^29, 41, 42^. IUPred (version 1.0) is based on statistical contact potentials and exclusively uses single sequences ^28, 43^. MD ( Meta-Disorder) ^42^ combines different original prediction methods through machine learning (neural network) with evolutionary profiles and predictions of solvent accessibility and protein flexibility. To some extent disorder is a gradual phenomenon, i.e. proteins may have more or less disorder ^44^. On the other hand, prediction methods distinguish between a 30-residue loop resembling “protein disorder” and another resembling a region with “regular structure” ^29^. Thus, protein disorder seems more a binary feature (it is there or not, or present/absent) than a gradual one ^25^. Unfortunately, no argument or data determines one single correct threshold for what constitutes present/absent for protein disorder. Typically, experts use a length threshold of the type: protein disorder is present when at least T consecutive residues in a protein are predicted to be disordered. If so, this protein is considered to contain a long region of disorder. More disorder in this model could imply, e.g. more than one region, or the entire protein. We analyzed many alternatives to choose the threshold for long disorder, and found most to be redundant. We included different views only if they provided relevant information. In particular, we largely focused on one threshold to define “long disorder”: **%long30**, is the percentage of proteins with at least one region of ≥30 consecutive residues predicted as disordered (alternatives were: %long50 and %long80, i.e. with length thresholds at ≥50 and ≥80, and completely disordered implying no region of 30 consecutive residues without any disorder).

### GO term enrichment

We applied BINGO (Biological Networks Gene Ontology ^44^, version 2.44) to identify the enrichment of GO (Gene Ontology) ^45^ terms in subsets of experimentally annotated proteins. We focused on “biological process” and “molecular function”. For two sets of proteins with annotated biological functions (more precisely: GO numbers) BINGO estimates to which extent their annotations differ in a statistically significant way. We visualized BINGO results with Cytoscape ^46^ platform (version 2.8). Our analysis focused on the hypergeometric test in BINGO, which accurately estimates p-values as it tests without replacement. Following the common, procedure for BINGO, we considered p-values >0.05 as significant ^46^. Testing multiple hypotheses may give many false positives (Type I error: incorrect rejection of true null hypothesis ^47, 48^). Using BINGO, we corrected for these through the Benjamini and Hochberg correction which provides strong control over the False Discovery Rate (FDR, expected proportion of erroneous null hypothesis rejections among all rejections ^48^).

## Results and discussion

### Duplications in response to high temperature reduce protein disorder

**Figure 1.**
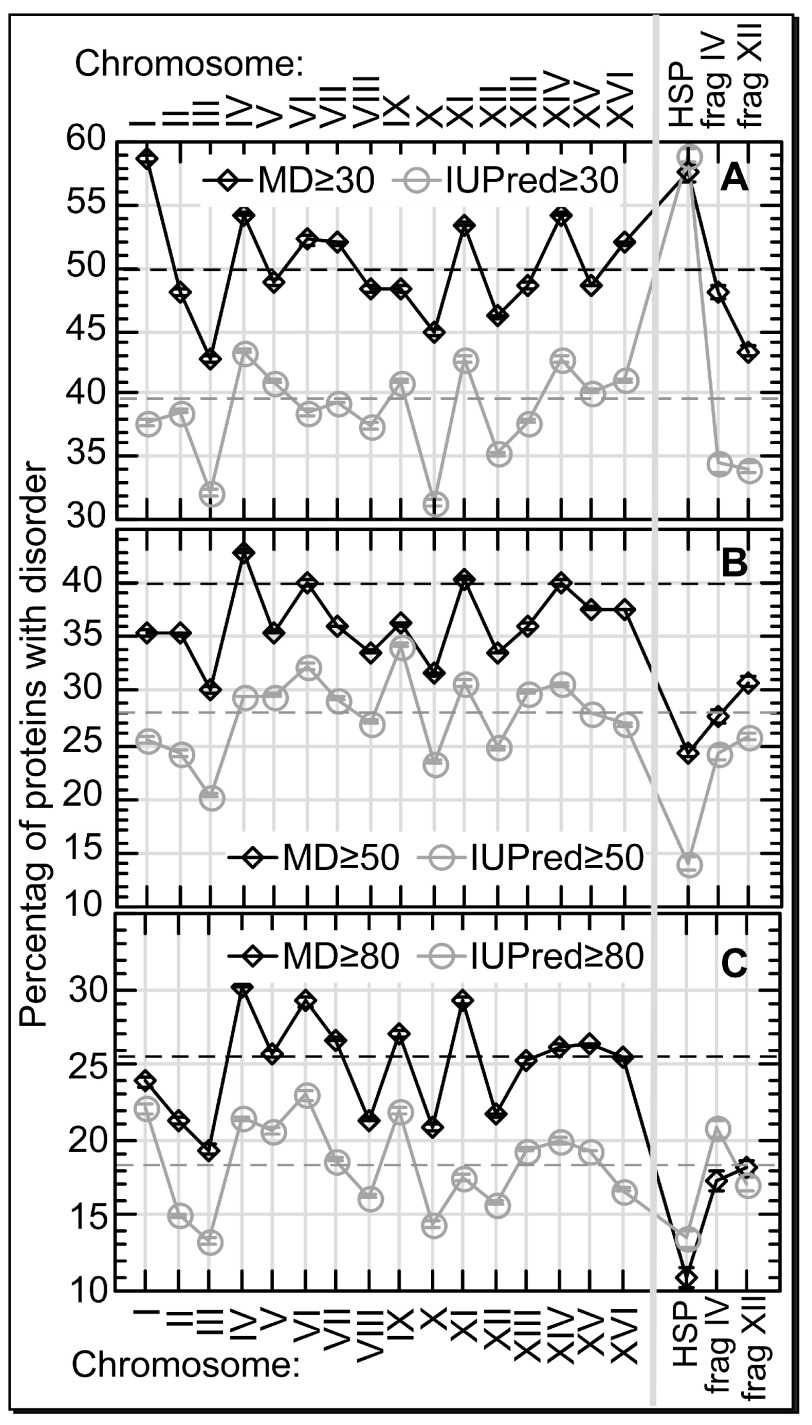
Protein disorder differs between yeast chromosomes. The composition of proteins with long regions of disorder (y-axes) differed significantly between the 16 chromosomes of *S. cerevisiae* (x-axes) and also for the set of HSPs. The three rightmost marks on the x-axes describe: HSPs and the disorder predictions for the HSR-related duplicated fragments on chromosome IV and chromosome XII ( frag IV and frag XII). The differences were similar for two different prediction methods (MD in black, IUPred in light gray), and for different thresholds with respect to the minimal length of a disordered region ( **A:** ≥30 consecutive residues predicted in disorder, **B**: ≥50, **C**: ≥80). Dashed horizontal lines mark the averages over all chromosomes. Error bars are too small to become visible on the scale chosen. The least disorder content was predicted for chromosome III and chromosome X. Overall, all duplications in response to heat shock treatment reduced the level of protein disorder in the offspring.

In response to high temperature yeast ( *S. cerevisiae*) duplicates the entire chromosome III (for brevity we use *chrN* to denote ‘yeast chromosome N’ with N as Roman numerals following convention) and fragments from chrIV and chrXII ^20^. The size of the 16 yeast chromosomes varies over six-fold ( Table S1). The average protein length is similar between the 16 chromosomes ( Figure S1, Table S1). The duplicated chrIII is the 3 ^rd^ smallest with 183 genes, of which 153 are mapped and 132 constitute “verified ORFs”. Fewer genes are encoded only by chrI with 90, and chrVI with 125 proteins ( Table S1). The relatively small number of genes on chrIII was one reason for choosing it as the first fully synthesized functional yeast chromosome ^49^. In contrast to protein length, the percentage of proteins with predicted long regions of disorder differed significantly between the 16 yeast chromosomes ( Figure 1).

The least protein disorder was predicted for chrIII and chrX ( Figure 1, Table S2). That means heat response duplicates one of the two chromosomes with the least disorder. In addition, the fragments of chrIV and chrXII that are duplicated along with the entire chrIII also clearly have less disorder than the chromosomes from which they were taken ( Figure 1). This enhances the effect of protein disorder reduction in response to high temperatures.

The other low-disorder option is chrX: Why not duplicate chrX in response to high temperatures? ChrX is more than twice as large as chrIII ( Figure S1). Thus duplicating chrX would “cost” twice as much. This might be prohibitive. ChrX might also not contain the cell activities important for coping with high temperature. Furthermore, as chrX and chrIII are similar in disorder content while chrX has twice the proteins of chrIII, the duplication of chrX would increase the overall level of proteins with disorder that might become unfolded and thereby “jam” cellular activity more than the duplication of chrIII.

Assume a certain amount of tolerable duplication were tolerable and that number were about 153 proteins (as for chrIII): where in the genome do we find a continuous stretch (within a chromosome) that has 153 proteins with the least disorder? Our results underscored the special role of chrIII ( Figure S2): only 3% of all continuous genome fragments with 153 proteins have as little disorder as chrIII (corresponding numbers for chrX: 5%; 29-protein fragment from chrIV: 52%; 64-protein fragment from chrXII: 10%). These figures demonstrate that the duplication of chrIII might be THE optimal choice for a simple way to duplicate 153 proteins with as little disorder as possible.

### Heat-shock proteins do not explain the temperature-related duplication

Our results explained why duplicating 150–200 proteins from another chromosome might have been potentially more damaging than the duplication of chrIII in response to high temperatures. In other words, our model might suggest why the duplication of this particular region is better than other duplications. However, what is the selective benefit from the proteins on chrIII? We expected to find the answer to this question in proteins that actively help with coping with heat stress. The immediate suspects are heat-shock proteins ( HSP) and the proteins known to interact with these HSPs ( HSP-binders). The known HSPs and HSP-binders scatter over all 16 yeast chromosomes ( Figure S3). All regions duplicated in response to heat shock contain only one known gene coding for HSPs ( *HSP30*) and one known HSP-binder ( *TAH1*). This implied that 1.3% of all known HSPs and HSP-binders were duplicated in an event that duplicated 0.5% of all genes, i.e. a 2.6-fold over-representation. This statistically insignificant the finding that fewer than 1 in 50 of all HSPs and HSP-binders are duplicated might still be scientifically significant if *HSP30* and *TAH1/HSP90* were outstandingly important proteins for the given conditions. However, this is not the case, at least not given what is currently known about *HSP30*. Furthermore, introducing an extra copy of *HSP30* into wild-type cells did not increase the ability of the cells to cope with high temperature (Dahan & Pilpel, personal communication).

The set of known HSPs ( Figure S3) slightly changed expression levels in response to heat stress during the fixation of the trisomy ^20^ only slightly but almost all HSPs were significantly up-regulated (arrows in Figure S3) when the “refined descendants” replaced the trisomy ^20^. This could imply that the duplicated genes are essential for survival under heat stress. Nevertheless, quite contrary to the naïve expectation, the HSPs and the HSP-binders by no means explained the heat-stress-specific duplications observed experimentally.

Incidentally, HSPs appeared particularly abundant in disorder regions of 30–50 consecutive residues ( Figure 1A, in particular for IUPred). It has previously been argued that such disorder is required for proper function of HSPs ^50^. In contrast, HSPs are depleted of longer disorder (>50; Figure 1B,C).

Overall, we argue that HSPs could have explained the duplication of many other chromosomes, possibly even better than that of chrIII. Therefore, this explanation is not specific. Thus, we conclude that the duplication of known HSPs and HSP-binding proteins did not explain why chrIII was specifically duplicated. Many HSPs and HSP-binders might remain unknown. However, we have no scientific ground to suspect that the fraction of the unknown HSPs differs between the chromosomes, i.e. that there are particular HSPs on chrIII that remain undiscovered.

### GO terms enriched for growth and reproduction in heat stress-duplication

Are any other proteins on chrIII important for growth under high temperature? Simply scanning GO ^45^ annotations is insufficient: the question is not whether proteins on chrIII have certain functions, but whether these are overrepresented enough to explain why chrIII and not the other two small chromosomes (chrVI or chrI) are duplicated in response to high temperatures. In order to address this question, we need a GO term enrichment analysis of the duplicated regions ^51^.

**Figure 2.**
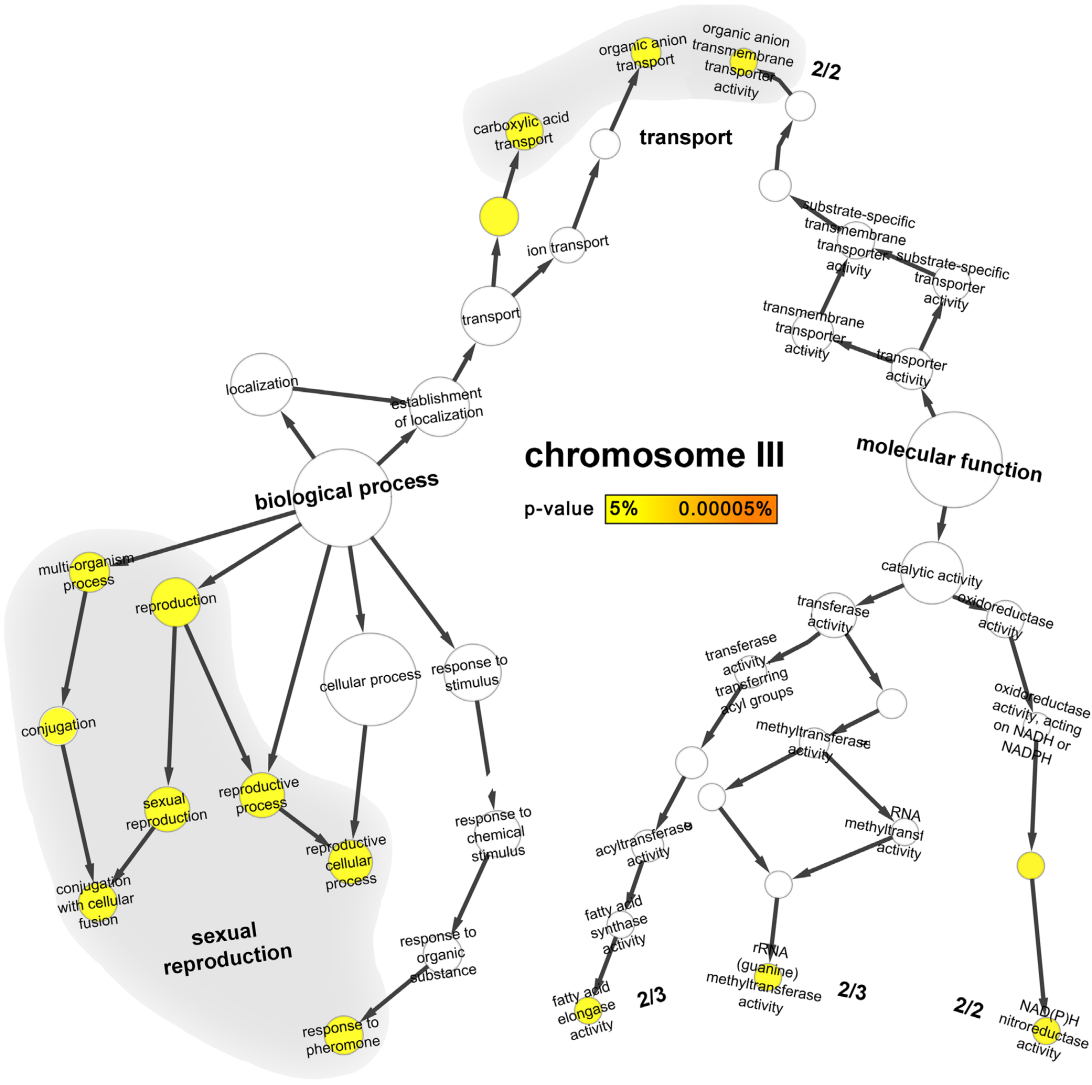
GO enrichment of sexual reproduction and nutrient uptake. The tree gives the complete set of all experimentally annotated GO-terms (Gene Ontology ^45^) for any of the proteins on chromosome III that describe *biological process* (left branch) and *molecular function* (right branch). The enrichment analysis ^51^ describes how much chrIII GO-terms are enriched with respect to all other GO-terms from yeast: all terms marked by yellow circles are significantly enriched. Sexual reproduction (7 GO-terms on chrIII) and transport (carboxylic acid and organic anion 4 GO-terms on chrIII) mapped to most overrepresented GO terms on this chromosome.

Growth and reproduction might be considered as the most important cell activities in the sense that the organism must grow and proliferate (cells that fail are not observed) even under stress. The GO enrichment analysis seemed to confirm this expectation ( Figure 2): the two most abundant GO terms in the heat stress-duplicated regions were those related to (i) sexual reproduction ( Figure 2 and Figure S2; “conjugation with cellular fusion”, “reproductive cellular process” and “response to pheromone”) and to (ii) sugar transport (hexose transport process as well as mannose, fructose and glucose transmembrane transporter activity; Figure S4).

The major energy source of yeast is sugar, in particular hexose monosaccharides (C6H12O6; e.g. glucose, fructose, mannose). These nutrient sugars are imported into the cell through hexose transporters, which are encoded by *HXT* genes ^52, 53^. The *HXT* yeast genes on the duplicated fragment of chromosome IV ( *HXT3*, *HXT6 and HXT7*) are almost five-fold over-represented with respect to random (yeast has 5667 N _genY_ genes, 243 N _genD_ are duplicated, 15 N _genHXT_ genes are in yeast; in a region with 243 N _genD_ genes we would find by chance 0.64 HXT genes in the duplicated regions p _chance_=N _genHXT_ *[N _genD_/N _genY_]). Two HXT genes on the duplicated chrIV fragment ( *HXT6* and *HXT7*) appear to encode high-affinity transporters required for growth at very low glucose concentrations (~0.1% ^54^), *i.e*. these two would become particularly important when yeast is cultured under glucose limitation ^54^. Interestingly, several works have detected duplication of these two genes ( *HXT6* and *HXT7*) in yeast populations evolving under low nutrient availability ^8, 55^. These numbers suggest that heat stress also puts strain upon obtaining the energy needed for growth and reproduction.

Sexual reproduction also appeared crucial for the survival of yeast cultured under heat stress ^56, 57^. Seven of the ten molecular functions to be significantly overrepresented in the heat stress-duplicated chrIII ( Table S3) by a standard GO-term enrichment analysis ^51^ are involved in reproduction. Three of these seven molecular functions are related specifically to sexual reproduction; the others pertain to general reproductive processes ( Figure 2). In particular, the reproduction-related processes involve cell fusion ( *FUS1* and *FIG2* ^58– 60^), pheromone response ( *STE50* which is also required for optimal invasive growth and hyperosmotic stress signaling ^61, 62^ and *NOT1* that is also involved in several RNA regulation levels ^63^), nuclear fusion, chromosome disjunction, nuclear segregation after mating ( *BIK1* which is involved in microtubule function during mitosis ^64, 65^), fusion of haploid nuclei during mating; *KAR4* or KARyogamy plays a critical role in the choreography of the mating response ^66^), cytokenesis (division of cytoplasma and plasma membrane of a cell and its separation into two daughter cells which is also relevant for asexual mitotic growth: *CDC10* ^67^), specification of the site where the daughter cell will form (relevant for budding and asexual growth, also referred to as axial bud selection) and in the developmental process in which the size of a cell is generated and organized (also referred to as morphogenesis: *CDC10* ^67– 69^). All these genes are also required for the correct localization of other proteins involved in cytokinesis and bud site selection ^67, 70– 73^. Other important processes and activities overrepresented on chrIII are related to the avoidance of oxidative stress (e.g. carboxylic acid transport – Figure 2 - which may be important for the survival since during the vegetative asexual reproduction cells were exposed to oxidative stress) and NAD(P)H nitro-reductase activity ( Figure 2). The only nitroreductase-related proteins in yeast – HBN1 and FRM2 ^74^ - are only on chrIII ( Table S4). The proteins involved in these two activities (carboxylic acid transport and NAD(P)H nitro-reductase activities) are also implicated in cellular detoxification ^75^, which is another task relevant for survival under stress.

All these data supported the view that chrIII is important for sexual reproduction. A seemingly convincing story, until we learned that the laboratory strains of yeast survived through asexual reproduction ^20^, *i.e*. apparently did not need what is so uniquely enriched in the heat stress duplication. The set of proteins known to be involved in reproduction on chrIII ( Table S3) had more disorder than the average for chrIII ( Figure S5). Some of these proteins with long disordered regions might not work correctly in heat.

Why duplicate proteins that fail? Not having found a convincing answer, we propose two conjectures: first, sexual reproduction might “frame” another cellular activity of the same protein that is more relevant to the growth conditions applied during the evolution in the laboratory experiment. For instance, *CDC10* is also required to maintain cell polarity (GO: 0030011), *BUD3* and *BUD5* are involved in axial cellular bud-site selection (GO: 0007120), *KCC4* a bud neck kinase involved in budding and cell bud growth (GO: 0007117) and *BIK1*, which is involved in microtubule function during mitosis. All of these activities are related to asexual reproduction. Our second proposition seems more far-fetched, namely that the set of proteins with the strongest GO-enrichment might have been duplicated coincidentally, *i.e*. the disorder-rich proteins related to sexual reproduction might have been duplicated because they happened to be on chrIII but not due their relevance for the survival in heat. If so, there must be something else we have not found yet on chrIII.

Several other processes were slightly enriched in the duplicated fragments with some relevance for yeast survival in heat but none of those gave a clear explanation ( Figure 2): (i) fatty acid elongase, (ii) rRNA (guanine) methyltransferase, and (iii) the importin-alpha export receptor activities. We analyzed these in detail. (i) Fatty acid elongase: currently, only three proteins are known to be involved in lengthening fatty acids; two of those (ELO2 and APA1; Table S3) are on chrIII. Fatty acid elongases are involved in sphingolipid biosynthesis. The sphingolipids are components of the cellular membrane and bioactive signaling molecules that contribute to heat tolerance as they are directly involved in organizational cellular structures (e.g. cell membrane) ^76^. (ii) rRNA methyltransferases: three yeast proteins are known to be involved in rRNA (guanine) methyltransferase activity; two of those ( *BUD23* and *SPB1*) are on chrIII ( Table S4). It is believed that the modification of ribonucleotides optimizes the rRNA structure and represents a way to expand the topological potentials of RNA molecules. It is possible that the loss of modification affects fine-tuning of ribosome function that could give rise to the pronounced cold-sensitivity ^77^. (iii) Importin-alpha nuclear export: two yeast proteins contribute toward the importin-alpha export receptor activity; one of those (MSN5) is in the duplicated fragment of chromosome IV. *MSN5* knockout mutants show a variety of phenotypes, including carbon-source utilization, defects and sensitivity to high concentrations of ions, severe heat shock, and high pH ^78^. Moreover, these mutants are partially sterile ^78^. Therefore, this protein appears necessary for cell survival, especially under extreme conditions.

Only one cellular activity related to tRNA synthase appeared overrepresented on the duplicated fragment of chrXII (DUS3 and DUS4 proteins; Table S7). In particular, to the tRNA dihydrouridine synthases, which are responsible for the reduction of the 5,6-double bond of a uridine residue on tRNA (one of the numerous modifications observed on tRNA cytoplasmatic ^79^). However, this particular finding appeared less relevant since the corresponding fragment was only duplicated in one of four growth experiments in response to high temperatures ^20^.

One crucial limitation for any functional enrichment study remains the incomplete experimental annotation even for an organism as intensively studied as yeast. It may be that all our speculation above missed the real causation because the functions of the proteins that are really relevant remain uncharacterized. Therefore, we complemented our analysis with one aspect of function for which we have a complete prediction, namely the prediction of sub-cellular localization of all yeast proteins. The experimental localization annotations for yeast are still cover at most 70% of all proteins ^80^. However, today’s top prediction methods, such as LocTree3, are very reliable ^80^ and can make crucial differences for comparing ‘complete’ data sets ^81^. We found nuclear proteins to be clearly depleted on chrIII (-4.6 percentage points with respect to the entire proteome; Figure S7A). Other abundant proteins found on chrIII were secreted (extra-cellular) or annotated as endoplasmic reticulum (ER) membrane proteins (each 3.2 percentage points higher than in the full yeast proteome). We also observed significantly more disorder in nuclear proteins (nuclear 77% vs. <40% for non-nuclear; Figure S8). This might explain the depletion of nuclear proteins on chrIII. While these findings were clear, they did not suggest a simple interpretation. The abundance of secreted proteins on chrIII (about 3.2 percentage points more on chrIII than in entire yeast; Figure S7A) implies that in the response to heat shock, more proteins are secreted into the ‘hot’ environment. Given the correlation between habitat and disorder ^37^, we expect that proteins are more likely to sustain high temperatures with less disorder. Unfortunately, a GO enrichment study of the secreted proteins also did not provide the answer we had been hoping for. However, the “secretome” alone could not explain the lower content of disordered proteins on chromosome III (disorder entire yeast-chrIII=50%-43%=7%>3% for secretome; Table 1 and Figure S7A).

### Proteins from chrIII less implied in overall PPI network

**Figure 3.**
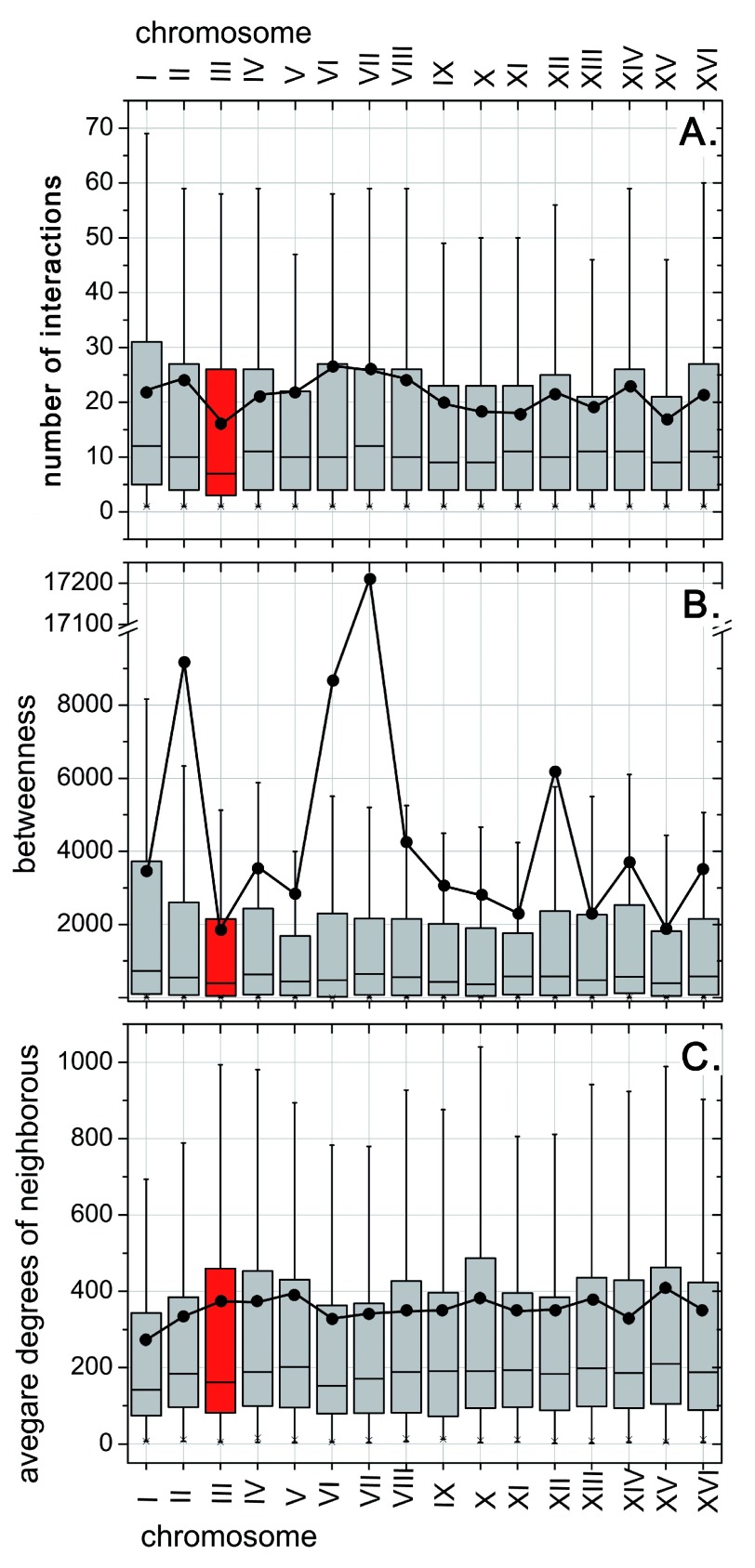
PPI network differs between yeast chromosomes. We began with the entire network of all PPIs with experimental annotations in yeast (Methods), and then differentially analyzed major network features: ( A) Degree: The number of PPIs per protein (degree) was minimal for the proteins from chrIII (box in red; lowest mean - black dot and lowest median - black line in the box. ( B) Betweenness: betweenness (number of times that a node acts as a bridge along the shortest path between two other nodes) was also lowest for chrIII. ( C) Average neighbor degree: plotting the average degree for all network neighbors of all proteins on chrIII ( *i.e.* all those proteins in direct PPI with proteins on chrIII), we observed a much less differentiated view. For this network feature, the proteins from chrIII had one of the highest means (black dot), but one of the lowest medians. Clearly, the proteins from the HSR-duplicated chromosome appeared less involved in the yeast network than expected by chance.

As proteins cannot be understood without also considering their networks of interaction, we compared the network of experimentally characterized PPIs between the entire yeast and those fragments that are duplicated in heat evolving populations. As for the differential analysis of any experimental annotation, the limitation of such an approach lies in the incompleteness of the experimental data. In all 16 chromosomes, the degree (number of interactions per protein) was lowest for chrIII (average=16±2; Figure 3A). A similar trend was observed for betweenness (number of times that a protein acts as a bridge along the shortest path between two other proteins: average=1800±300; Figure 3B). Furthermore, chrIII is one of the chromosomes with the largest mean value for the average neighbor degree (average=380±40; Figure 3C). Our network analyses confirm chrIII as a good choice for a first line of defense against high temperature because the proteins encoded on this chromosome play less essential roles for the overall PPI network. However, once again, this portrays the duplication as a solution with least possible damage without positively suggesting causation.

## Conclusions

Organisms can duplicate the whole genome or particular chromosomes (aneuploidy) in response to sudden dramatic changes in the environment. As such coarse-grained major changes are costly, aneuploidy tends to give way to more fine-tune focused solutions that require many generations to evolve. The entire chromosome III and two fragments from chromosomes IV and XII in a culture of budding yeast ( *S. cerevisiae*) were duplicated as a “transient evolutionary solution” in response to high temperature - a “transition” that fostered the survival of between 400 and 2,000 generations. Here, we reported that while the proteins on all 16 main chromosomes from yeast have similar length, they differ substantially in the fraction of proteins with long regions predicted to contain protein disorder (≥30–80 consecutive residues predicted as disordered by IUPred and MD). We found the regions duplicated under heat stress depleted of predicted disorder. In fact, chromosome III was one of the two chromosomes with the least disorder ( Figure 1). The other (chromosome X) is twice as large, *i.e*. would cost twice to duplicate. Decreasing the overall content in protein disorder is likely an important strategy to protect against heat stress. A detailed analysis of the experimentally characterized PPI network in yeast revealed the duplicated proteins to be connected less than average ( Figure 3). The PPI analysis, therefore, added to the explanation that the duplication causes minimal damage. However, why did the duplication create an advantage under heat stress? Surprisingly, we found no sustained evidence for a significant over-representation of HSPs in the duplication i.e. of proteins that usually help out under such stress. Instead, a Gene Ontology (GO) enrichment analysis suggested that the duplicated regions were enriched in processes related to reproduction and to the import of nutrients ( Figure 2). The enrichment was strongest for proteins related to sexual reproduction although the heat stress survival was maintained through budding, *i.e.* through asexual reproduction. Nevertheless, the set of GO enriched proteins appeared so important that they were duplicated although high in disorder. This might point to where the explanation for the duplication might be found. Overall, our data suggested a very simple algorithm: identify the region with lowest protein disorder that is large enough, yet not too large and duplicate it along with possibly other fragments that are also depleted of disorder in order to cope with heat stress.

## Supplementary material

Supplementary material for Vicedo *et al.*, 2015 'Protein disorder reduced in *Saccharomyces cerevisiae* to survive heat shock'.

*Table of Contents for Supplementary Material*

Figure S1: Number of genes per chromosome in *Saccharomyces cerevisiae* (yeast).

Figure S2: Fragments with less disorder than proteins duplicated during heat shock response (HSR).

Figure S3: Distribution of Heat Shock proteins (HSP) along chromosomes.

Figure S4: Complete set of known GO (Gene Ontology) terms for a fragment of the HSR-duplicated chromosome IV.

Figure S5: Disordered proteins differentiate by chromosomes and roles.

Figure S6: Disorder difference between chromosome III proteins and their paralogs.

Figure S7: Distribution comparison of chromosome III proteins and the complete yeast proteome across localization classes.

Figure S8: Distribution of disordered/ordered nuclear proteins and other yeast proteins.

Table S1: General information about yeast chromosomes.

Table S2: Disorder abundance content.

Table S3: Overrepresented GO terms for molecular function analysis of chromosome III.

Table S4: Overrepresented GO terms for biological process analysis of chromosome III.

Table S5: Overrepresented GO terms for molecular function of the duplicated fragment of chromosome IV.

Table S6: Overrepresented GO terms for biological process of the duplicated fragment of chromosome IV.

Table S7: Overrepresented GO terms for biological process of the duplicated fragment of chromosome XII.

Table S8: Heat shock proteins distribution on chromosomes.
