# Supplementary material for: Protein disorder reduced in Saccharomyces cerevisiae to survive heat shock
Source: F1000Res. 2015 Nov 6;4:1222. [Version 1] doi: 10.12688/f1000research.7178.1 (PMC4670006; doi:10.12688/f1000research.7178.1)
Supplement: Supplementary file 1 [file f1000research-4-7734-s0000.tgz › 79ffb717-71b1-4967-aa63-f1e0471eb49c.docx]

Supplementary material for:

Protein disorder reduced in *Saccharomyces cerevisiae* to survive heat shock

Esmeralda Vicedo, Zofia Gasik, Yu-An Dong, Tatyana Goldberg & Burkhard Rost

# Table of Contents for Supplementary Material

**Fig. S1: Number of genes per chromosome in *Saccharomyces cerevisiae* (yeast).**

**Fig. S2 Fragments with less disorder than proteins duplicated during heat shock response (HSR).**

**Fig. S3: Distribution of Heat Shock proteins (HSP) along chromosomes.**

**Fig. S4: Complete set of known GO (Gene Ontology) terms for a fragment of the HSR-duplicated chromosome IV.**

**Fig. S5: Disordered proteins differentiate by chromosomes and roles*.***

**Fig. S6: Disorder difference between chromosome III proteins and their paralogs.**

**Fig. S7: Distribution comparison of chromosome III proteins and the complete yeast proteome across localization classes.**

**Fig. S8: Distribution of disordered/ordered nuclear proteins and other yeast proteins.**

**Table S1: General information about yeast chromosomes.**

**Table S2: Disorder abundance content.**

**Table S3: Overrepresented GO terms for molecular function analysis of chromosome III.**

**Table S4: Overrepresented GO terms for biological process analysis of chromosome III.**

**Table S5: Overrepresented GO terms for molecular function of the duplicated fragment of chromosome IV.**

**Table S6: Overrepresented GO terms for biological process of the duplicated fragment of chromosome IV.**

**Table S7: Overrepresented GO terms for biological process of the duplicated fragment of chromosome XII.**

**Table S8: Heat shock proteins distribution on chromosomes.**

# Short description of Supplementary Material

The Supplementary material is not essential to support any of the major results and points of our manuscript. Instead, we provide additional data to support some of the minor.

**Fig. S1:**  presents the distribution of the percentage of genes mapped to each of the 16 chromosomes of yeast and the distribution of the average protein length on each chromosome.

**Fig. S2:** shows the percentage of regions on the 16 nuclear chromosomes which contain more disordered regions of the same length as the regions duplicated during the heat shock response (HSR).

**Fig. S3:** shows the distribution of heat shock proteins (HSP) and their interactors among 16 nuclear chromosomes in yeast.

**Fig. S4:** shows mapped molecular function and biological process GO terms to genes/proteins of fragments on chromosome IV.

**Fig. S5:** shows the content of disordered proteins differentiated by chromosomes and their roles in yeast (*i.e*. reproduction proteins, no reproduction proteins and HSP proteins).

**Fig. S6:** shows the comparison of the disorder content of yeast proteins from chromosome III and their paralog proteins from other chromosomes.

**Fig. S7:** shows the distribution of proteins across 15 predicted sub-cellular localization compartments of the complete proteome of yeast and chromosome III. It also shows the distribution of predicted ordered and disordered proteins for both, *i.e.* the complete proteome and chromosome III.

**Fig. S8:** shows the analysis of order and disorder in nuclear and non-nuclear proteins.

**Table S1:** provides general information about yeast chromosomes (length of chromosome in bp, number of encoded genes, etc.).

**Table S2:** provides the protein disorder content on XVI autosomal chromosome using the predictors MetaDisorder (MD) and IUPred. A protein is considered as disordered when it contains a region of >30/50/80 consecutive disordered residues.

**Table S3, Table S4, Table S5, Table S6 and Table S7:** lists of overrepresented GO terms (from the molecular function and biological process ontologies) for proteins from chromosome III and from duplicated regions of chromosomes IV and XII.

**Table S8:** lists all heat-shock proteins with their corresponding localization on yeast chromosomes and expression levels.

# Supplementary Material: Figures

### Fig. S1

_
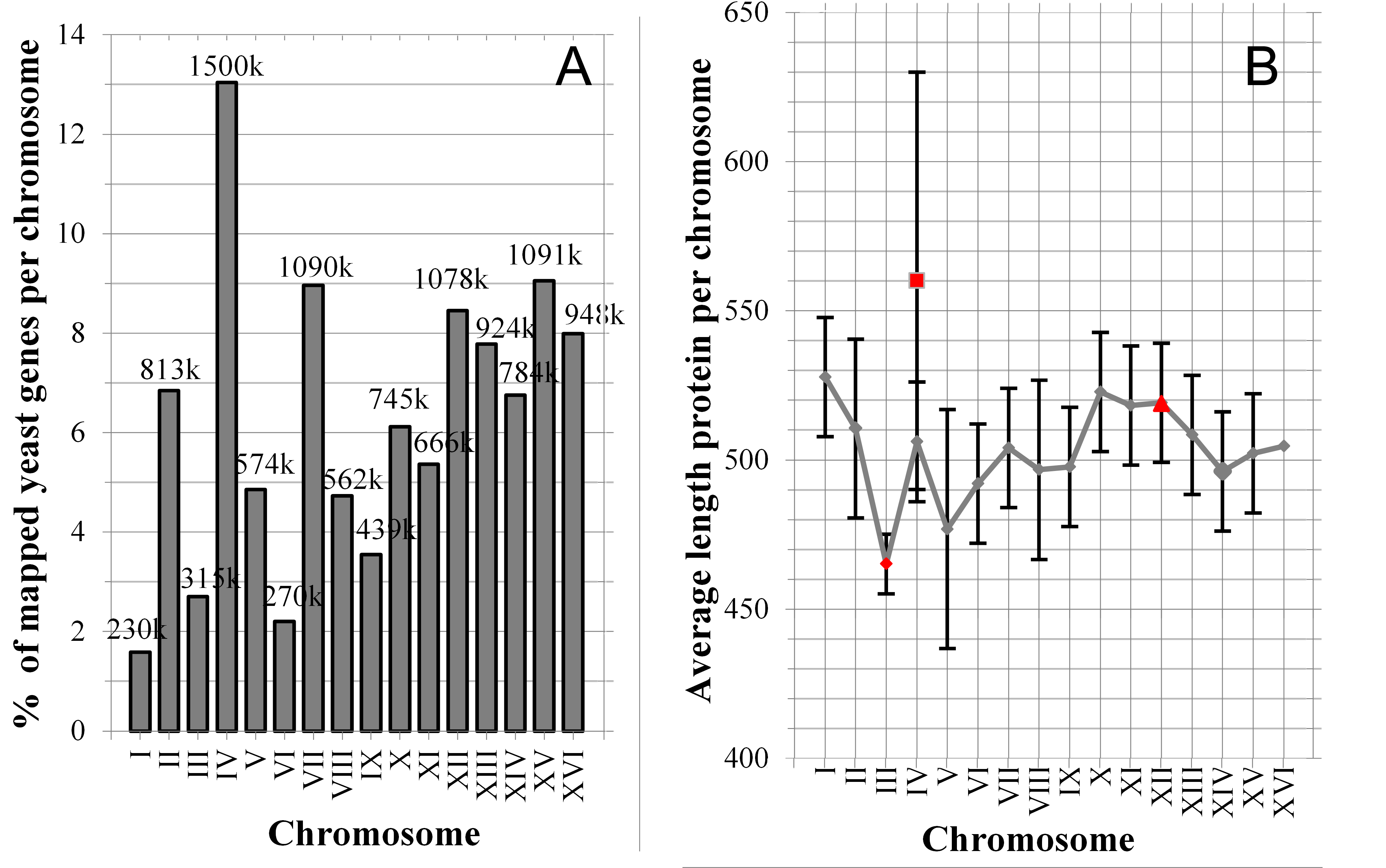
_

**Fig. S1: Number of genes per chromosome in *Saccharomyces cerevisiae* (yeast).** (A) Distribution of the percentage of genes mapped to each of 16 chromosomes in yeast (*Saccharomyces cerevisiae*). Chromosome IV is the largest, chromosome I the smallest. The numbers on top of the bars mark the length of a chromosome in kilo-base pairs (k). (B) Distribution of the average protein length on each chromosome. Red dots mark the duplicated chromosome III and fragments of chromosomes IV and XII. There is no significant difference in protein lengths.

### Fig. S2


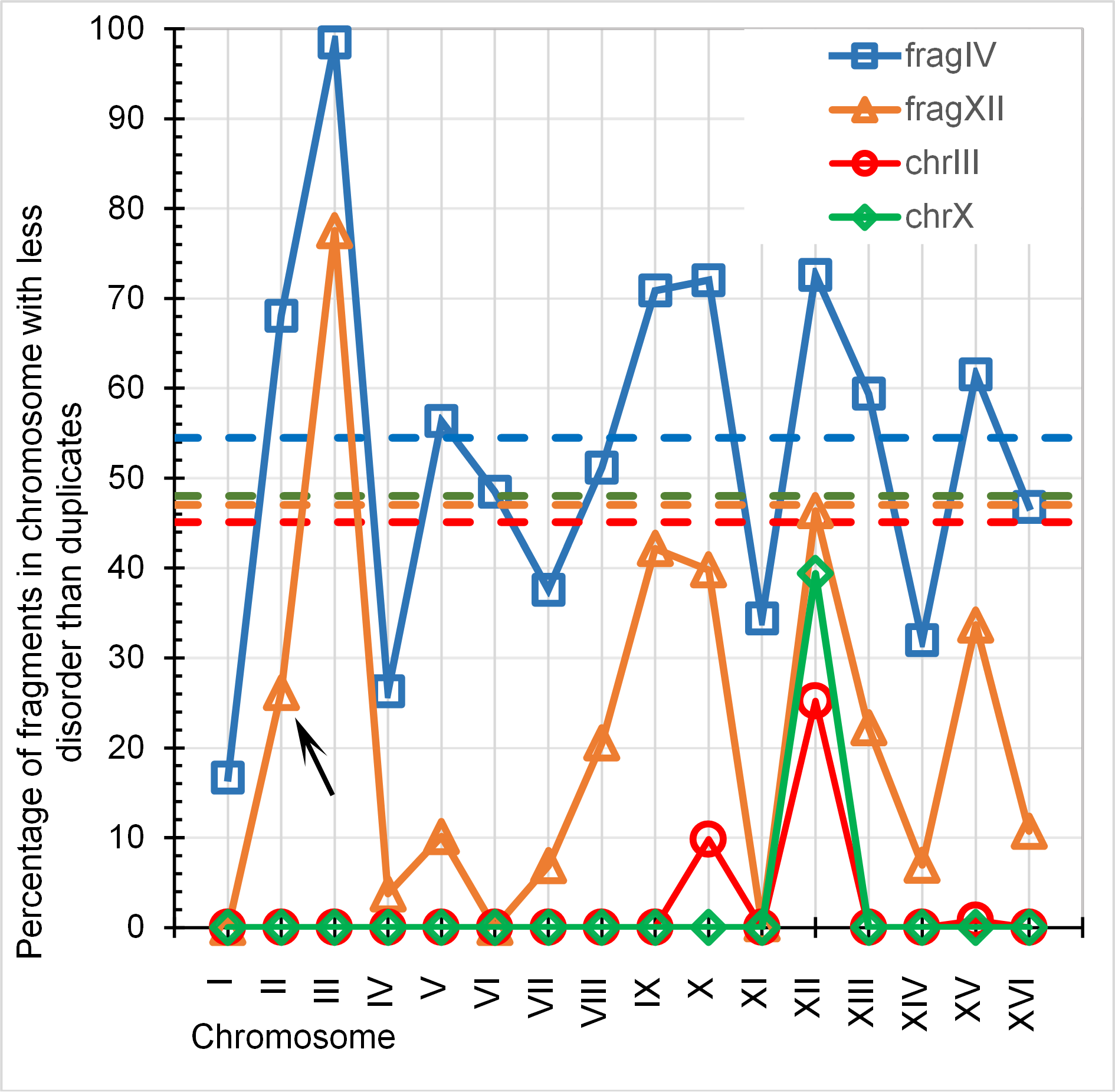


**Fig. S2: Fragments with less disorder than proteins duplicated during heat shock response (HSR).** First, we estimated the number of proteins, encoded by the duplicated chromosome III and the fragments of chromosomes IV and XII, which are involved in the heat shock response (HSR). We found 153 proteins on chromosome III, 29 proteins on the fragment of chromosome IV and 64 proteins on the fragment of chromosome XII. We estimated the disorder content in these sets of proteins: 55% in proteins of fragment of chromosome IV (blue dashed line), 47% in proteins of fragment of chromosome XII (orange dashed line) and 45% proteins of chromosome III (red dashed line). We also estimated the disorder content for all 347 proteins of chromosome X, which is the non-duplicated with less disorder content and its proteins are known to not being involved in HSR. The disorder content was 48% (green dashed line) for proteins of chromosome X. Then, we screened all chromosomes for stretches encoding 153, 29, 64 and 347 proteins to measure their disorder content and compare it to the disorder content in chromosomes III, X and the fragments of chromosomes IV and XII. For example, chromosome II contains 388 proteins, so we measured the disorder content in 325 stretches encoding 64 proteins (as this is the number of HSR proteins encoded by chromosome IV) to compare their disorder content to that in proteins of chromosome IV. We found that proteins encoded by 68% of regions of chromosome IV have less disorder than 64 proteins of chromosome IV (black arrow).

### Fig. S3


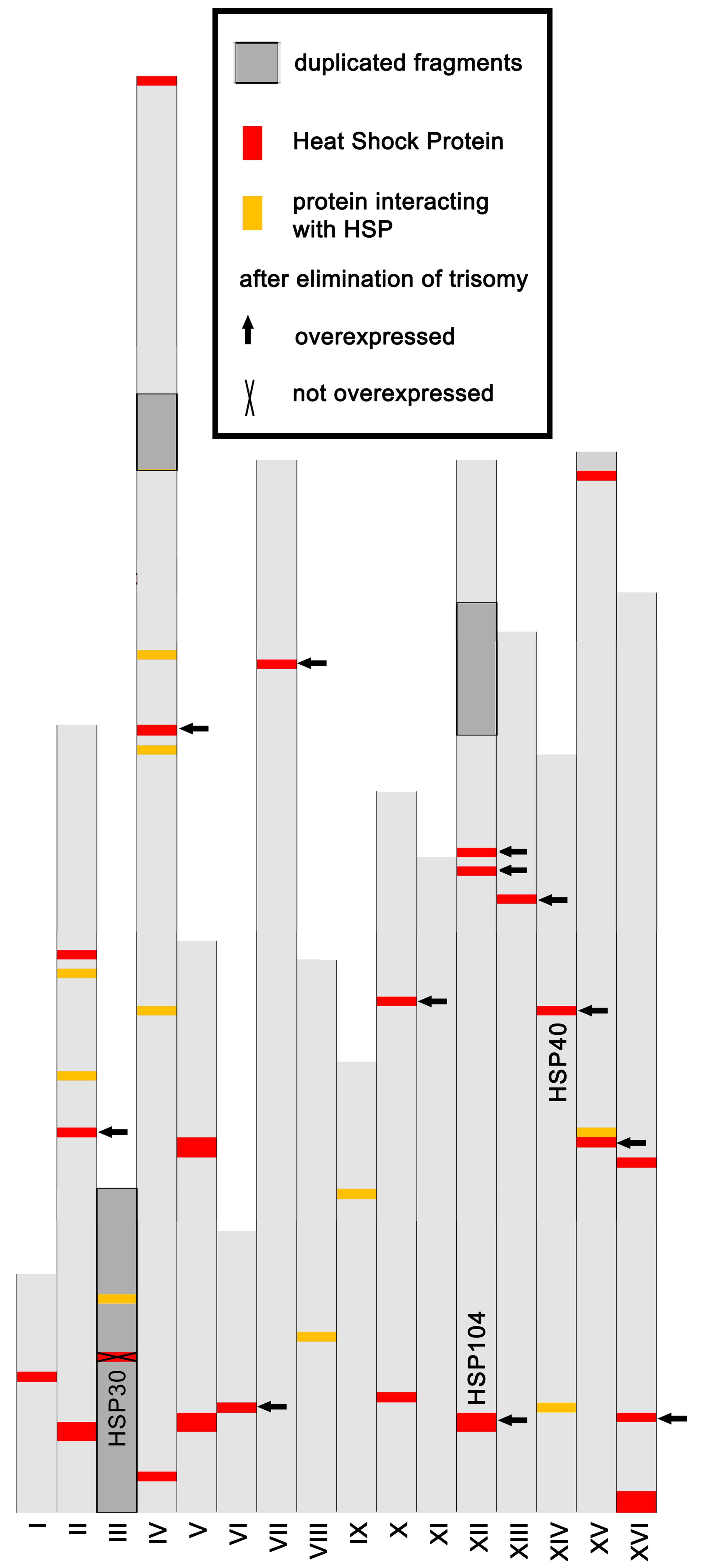


**Fig. S3:** **Distribution of Heat Shock proteins (HSP) along chromosomes.**

The duplicated chromosome III (III) and the duplicated fragments of chromosomes IV (IV) and XII (XII) (dark grey) contain only one known heat shock protein (HSP; on chromosome III, marked in red: HSP30) and one protein known to interact with it (also on chromosome III: marked in yellow: TAH1). Nevertheless, the HSP and HSP-interactors appear numerically over-represented by a factor of two on the duplicated genes on chromosome III, as 1.3% of the HSP proteins are duplicated compared to 0.7% of all genes. Given the large margin of missing experimental knowledge about HSP proteins, a single experimental paper might alter these numbers. HSP30 encodes a highly hydrophobic integral membrane protein. Its expression is just detectable in exponentially growing yeast cells and increases dramatically in level in heat shock response (HSR) and in response to treatment with ethanol and sorbate. One aspect of function of such a system might be that it minimizes the effects of stress on the membrane. There are preliminary indications for this protein having several roles at the plasma membrane [2-5]. Overall, known HSPs (marked with crosses and arrows) change their expression levels modestly during the fixation of the trisomy but almost all of them were significantly up-regulated when the trisomy was replaced by the refined descendants (subset of genes retain elevated expression). This could imply that the duplicated genes are essential for the survival of the yeast under heat shock.

### Fig. S4

**
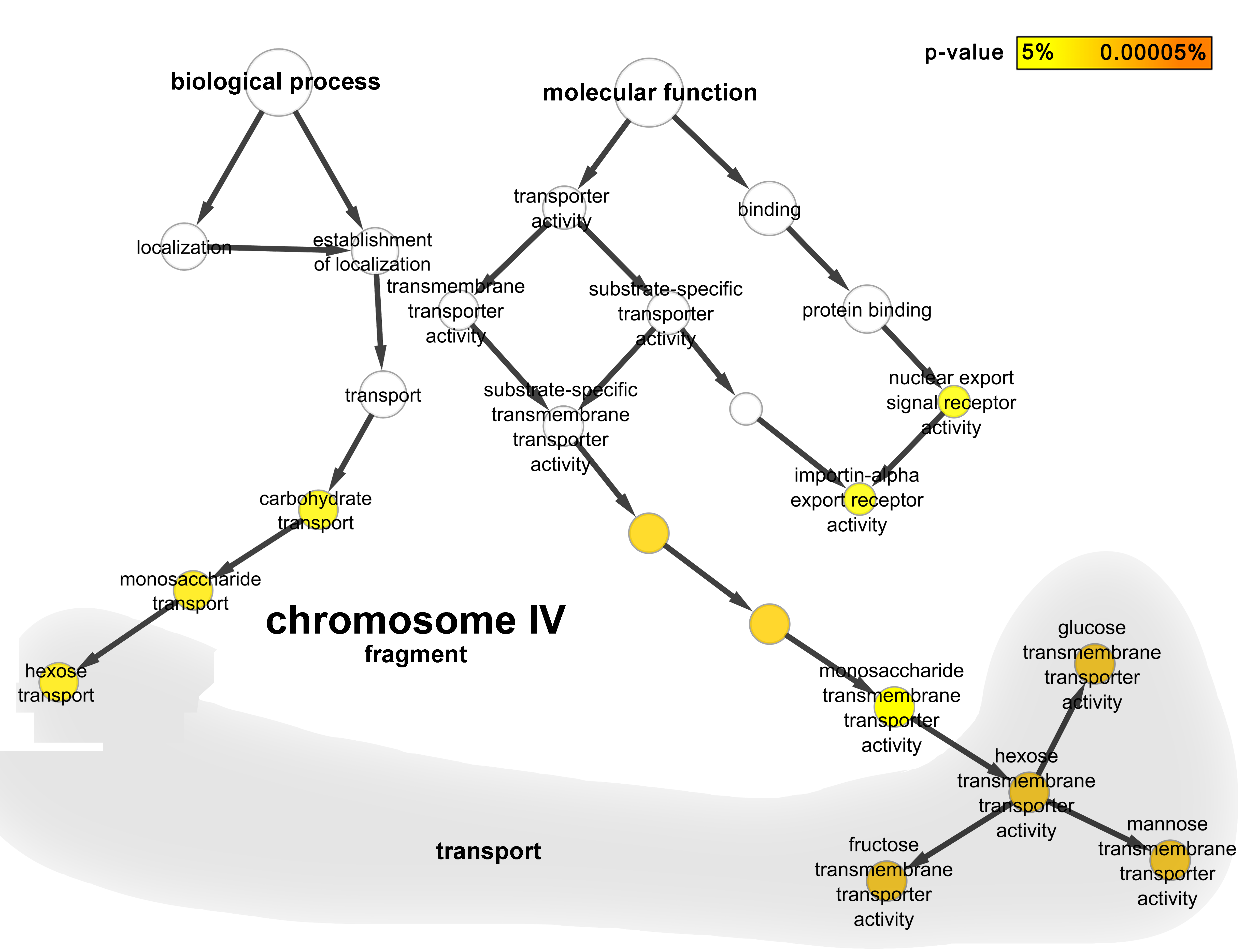
**

**Fig. S4: Complete set of experimentally known GO (Gene Ontology) terms for a fragment of the HSR-duplicated chromosome IV.** The molecular function and the biological process ontologies are represented together to provide an overview over GO terms mapped to genes/proteins fragments of chromosome IV (ChrIV). A standard GO term enrichment analysis [1] suggests that most of these terms are significantly overrepresented (yellow and orange circles). Hexosa transport is the process involving most overrepresented GO terms (9). In nutrient-rich environments, hexose monosaccharides (C6H12O6; e.g. glucose, fructose, mannose) present the most common energy source for yeast. The first step to get to this source is the transport across the plasma membrane via diffusion by a group of membrane-spanning proteins named hexose transporters encoded by HXT genes. Of these proteins, 13% are involved in the transport of hexose; they are on the duplicated fragment of ChrIV (HXT3, HXT6 and HXT7).

### Fig. S5


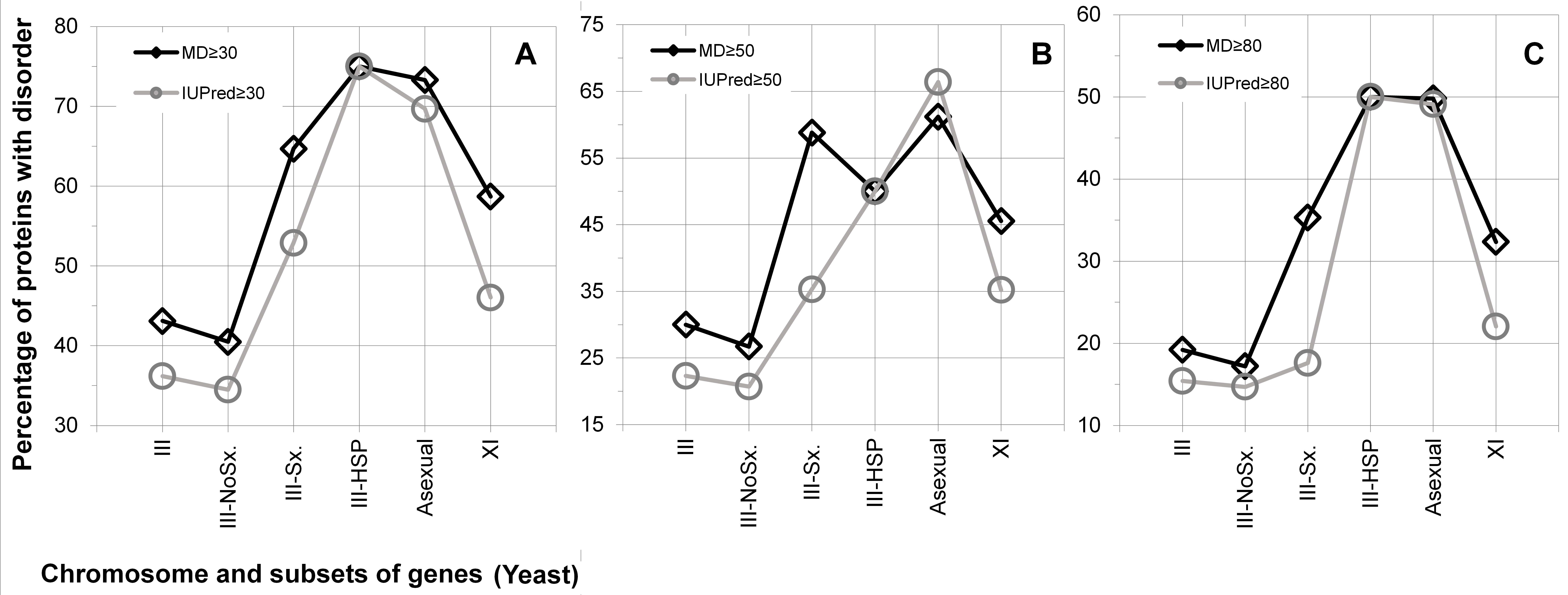


**Fig. S5: Disordered proteins differentiate by chromosomes and roles*.*** All proteins from chromosome III are grouped according to their known role in reproduction (III-NoSx: proteins that are not known to be involved in sexual reproduction, III-Sx: proteins that are involved in sexual reproduction) and according to the known annotations as Heat Shock Proteins (HSPs: III-HSP). The content in proteins with long disorder (A: ≥30, B: ≥50, and C: ≥80 consecutive residues predicted to be disordered) differed substantially between these sets: proteins not involved in sexual reproduction (III-NoSx) had the lowest amount of disorder; in fact, even less than the overall level for chromosome III (leftmost point marked as “III”). Proteins involved in asexual reproduction (“Asexual” in all chromosomes) had even more disorder than the overall level for chromosome XI, which was one of the chromosomes with the highest protein disorder content. The same trend was observed for III-HSP-related proteins (only those that are localized on chromosome III, leaving only four proteins).

### Fig. S6


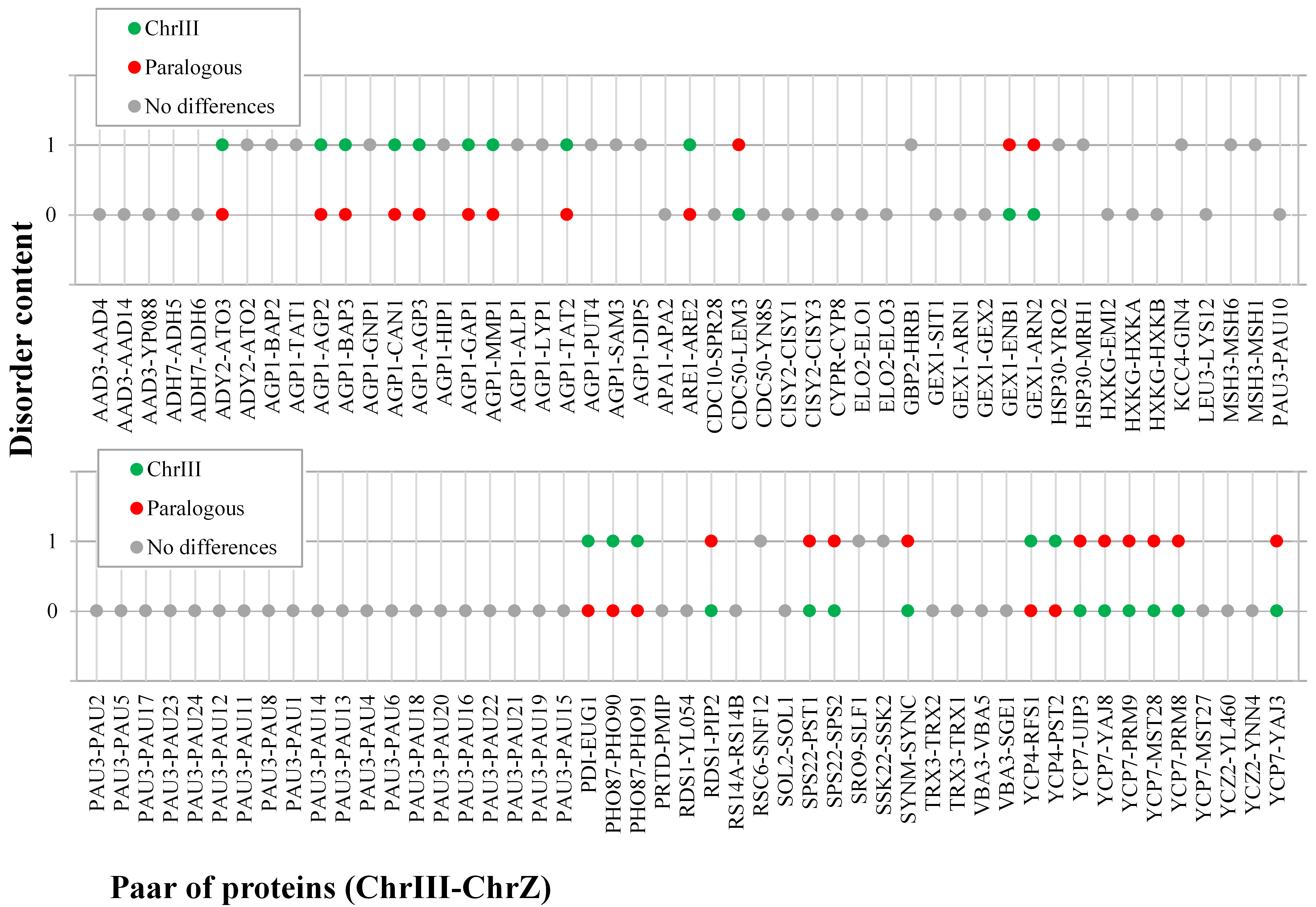


**Fig. S6: Disorder difference between chromosome III proteins and their paralogs.**

We analyzed all proteins from chromosome III (chrIII) for which we found a paralog on another chromosome with respect to their disorder content. Given are the identifiers of all those pairs. Green points mark examples for which the disorder was higher in the chrIII version (first name in each pair, 14% of all pairs), red points mark those for which the outside-III paralog (second name in each pair, 13% of all pairs) had more disorder, gray points mark those without difference in disorder (73% of all pairs). The protein disorder content for the two analyzed subsets of paralogs is similar (subset chrIII: 34.3%; paralogs to chrII: 32.7%), but lower than that of chrIII (43.1%).

### Fig. S7

**
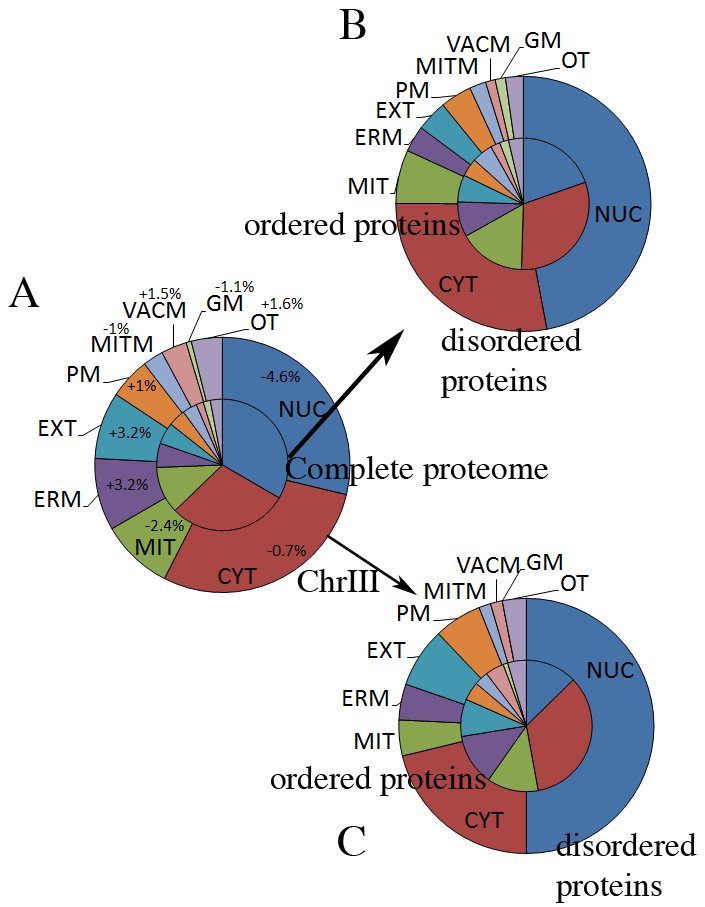
**

**Fig. S7: Distribution comparison of chromosome III proteins and the complete yeast proteome across localization classes.** Three perspectives a differential views are given through the inner and outer pies: **A**: all proteins in yeast (inner pie) vs. those in chromosome III (outer pie), **B**: proteins in the entire yeast with long disorder (outer) vs. without (inner), and **C**: proteins on chromosome III with long disorder (outer) vs. without (inner). We predicted the localization of proteins across 15 different sub-cellular compartments using LocTree3 [6]. **(A)** Comparison of the distribution across the compartments for all proteins in yeast (inner pie, 5667 proteins) and for proteins of chromosome III (outer ring, 153 proteins). For each compartment we provide the difference in two distributions. **(B)** Comparison of the distribution of predicted (method %long30 and MD) ordered (inner pie) and disordered proteins (outer ring) across different localization compartments in the entire proteome of yeast and **(C)** in the proteins encoded by chromosome III. While there is no significant difference between the distributions of order and disorder in the sets of proteins of the entire yeast proteome and of chromosome III, there is a difference between the distribution of order and disorder within each of the sets. Namely, in both sets about 50% of all ordered proteins are predicted to reside in the nucleus and the cytoplasm. However, the fraction of disordered proteins predicted to reside in the same two compartments is 75%. These differences could be explained through their function (transcription and protein remodeling, as well as cell signaling).

Abbreviations: NUC, nucleus; CYT, cytoplasm; MIT, mitochondria; ERM, Endoplasmic Reticulum membrane; EXT, extra-cellular; PM, plasma membrane; MITM: mitochondria membrane; VACM, vacuole membrane; GM, Golgi apparatus membrane; OT, other (including Golgi apparatus, Endoplasmic Reticulum, vacuole, peroxisome, peroxisome membrane and nucleus membrane.

### Fig. S8

**
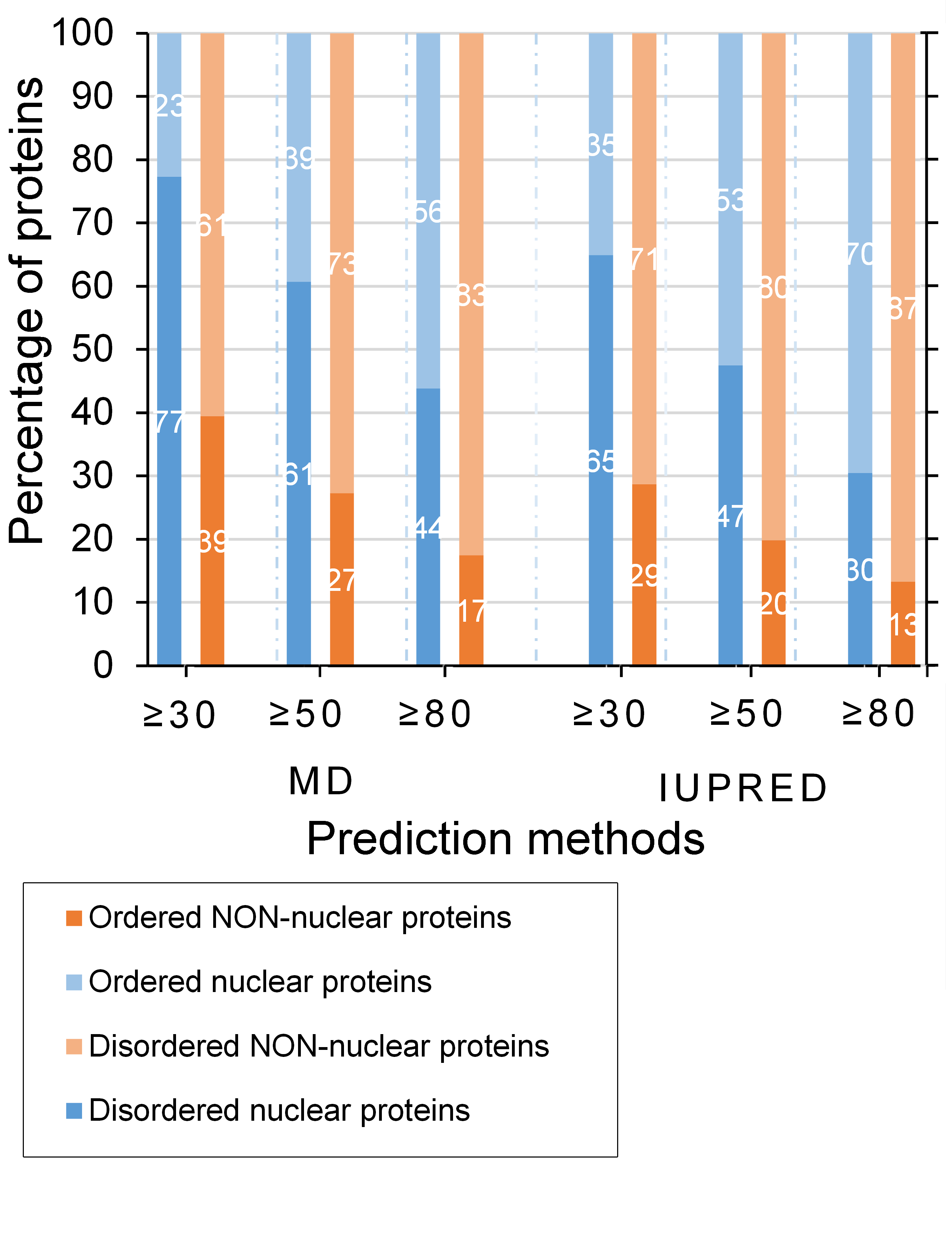
**

**Fig. S8: Distribution of disordered/ordered nuclear proteins and other yeast proteins.** Proteins predicted as nuclear are classified as disordered (dark blue) or ordered (light blue) depending if they contain a long disordered region (>30/>50/>80 consecutive amino acids predicted to be disordered). Proteins from the remaining cellular compartments, such as cytoplasm, membrane (cytoplasmic and nuclear) and secreted are grouped to be non-nuclear (disordered as dark orange, ordered as light orange). The content of disordered proteins in the nucleus is clearly higher than in the non-nuclear compartments.

# Supplementary Material: Tables

### Table S1: General information about yeast chromosomes.

| **chr. ^a^** | **length ^b^ [bp]** | **n. of genes ^c^** | **verified ORFs ^d^** | **mapped genes ^e^** | **% mapped genes ^f^** | **length ^g^ [aa]** | **min. length ^h^** | **max. length ^i^** |
| --- | --- | --- | --- | --- | --- | --- | --- | --- |
| **I** | 230,195 | 117 | 75 | 90 | 77% | 528 ± 40 | 55 | 1537 |
| **II** | 813,137 | 456 | 346 | 388 | 85% | 511 ± 20 | 65 | 3092 |
| **III** | 315,344 | 183 | 132 | 153 | 84% | 465 ± 30 | 40 | 2167 |
| **IV** | 1,522,191 | 836 | 659 | 739 | 88% | 506 ± 10 | 25 | 3268 |
| **V** | 574,860 | 324 | 235 | 275 | 85% | 477 ± 20 | 34 | 2167 |
| **VI** | 270,148 | 141 | 99 | 125 | 89% | 492 ± 40 | 28 | 2278 |
| **VII** | 1,090,936 | 583 | 450 | 508 | 87% | 504 ± 20 | 66 | 2672 |
| **VIII** | 562,638 | 321 | 232 | 268 | 83% | 497 ± 20 | 58 | 3744 |
| **IX** | 439,885 | 241 | 172 | 201 | 83% | 498 ± 30 | 99 | 2376 |
| **X** | 745,440 | 398 | 304 | 347 | 87% | 523 ± 20 | 39 | 2470 |
| **XI** | 666,448 | 348 | 280 | 304 | 87% | 518 ± 20 | 39 | 4092 |
| **XII** | 1,078,172 | 578 | 416 | 479 | 83% | 519 ± 20 | 56 | 4910 |
| **XIII** | 924,430 | 505 | 386 | 441 | 87% | 508 ± 20 | 36 | 1785 |
| **XIV** | 784,328 | 435 | 330 | 383 | 88% | 496 ± 20 | 38 | 2233 |
| **XV** | 1,091,282 | 598 | 462 | 513 | 86% | 502 ± 20 | 25 | 3079 |
| **XVI** | 948,061 | 511 | 395 | 453 | 89% | 505 ± 20 | 50 | 2489 |
| **IV fr.** | 66,475 | 30 | 27 | 29 | 97% | 560 ± 70 | 72 | 1514 |
| **XII fr.** | 127,467 | 64 | 63 | 64 | 98% | 519 ± 50 | 56 | 1770 |
| **total** |  | 6575 | 4973 | 5667 |  |  |  |  |

a. Chr. marks the number of a studied chromosome or a fragment (fr.) from *yeast.*

b. Length is the number of base pairs constituting a chromosome. Information obtained from http://www.yeastgenome.org/. Chromosome IV is the largest and chromosome I is the shortest.

c. N. of genes is the number of genes encoded on a chromosome. Information obtained from http://www.yeastgenome.org/. Chromosome IV is the one with the largest number of genes and chromosome I is the one with the fewest. This is related to the length of chromosomes.

d. Verified ORFs mark the number of expressed Open Reading Frames in yeast, taken from http://www.yeastgenome.org/. This value correlates with the length and the number of genes.

e. Mapped genes mark the number of genes on a certain chromosome that can be mapped to UniProt proteins.

f. % mapped genes is the fraction of genes on a certain chromosome that can be mapped to UniProt proteins.

g. Length marks the average length of proteins on a specific chromosome. Chromosomes III and V are the chromosomes whose genes are coding the shortest proteins. Chromosome XII and the fragment of chromosome IV code for the longest proteins.

h. Min. length marks the shortest protein encoded by the genes on a specific chromosome. Chromosomes IV and XV codify shortest proteins and chromosome IX largest.

i. Max. length marks the largest protein codified by the genes on a specific chromosome. Chromosome XII codifies a largest protein and chromosome I a shortest protein.

_________________________________________________________________

### Table S2: Disorder abundance content.

|  | **MD ^b^** | | | **IUPred ^b^** | | |
| --- | --- | --- | --- | --- | --- | --- |
| **chr. ^a^** | **>30 ^c^** | **>50 ^c^** | **>80 ^c^** | **>30 ^c^** | **>50 ^c^** | **>80 ^c^** |
| **I** | 64.4 ± 0.5 | 37.8 ± 0.5 | 28.9 ± 0.4 | 43.3 ± 0.5 | 34.4 ± 0.5 | 24.4 ± 0.4 |
| **II** | 52.7 ± 0.2 | 39.5 ± 0.2 | 25.3 ± 0.2 | 42.8 ± 0.3 | 29.1 ± 0.2 | 19.8 ± 0.2 |
| **III** | 45.1 ± 0.4 | 35.3 ± 0.4 | 21.6 ± 0.3 | 37.3 ± 0.4 | 24.2 ± 0.3 | 15.7 ± 0.3 |
| **IV** | 57.2 ± 0.2 | 46.4 ± 0.2 | 32.3 ± 0.2 | 46.8 ± 0.2 | 34.1 ± 0.2 | 24.2 ± 0.2 |
| **V** | 54.2 ± 0.3 | 39.6 ± 0.3 | 29.1 ± 0.3 | 43.3 ± 0.3 | 33.8 ± 0.3 | 24.7 ± 0.3 |
| **VI** | 53.6 ± 0.4 | 42.4 ± 0.4 | 30.4 ± 0.4 | 43.2 ± 0.4 | 32.8 ± 0.4 | 24.0 ± 0.4 |
| **VII** | 53.7 ± 0.2 | 40.8 ± 0.2 | 30.1 ± 0.2 | 43.5 ± 0.2 | 30.3 ± 0.2 | 22.4 ± 0.2 |
| **VIII** | 51.9 ± 0.3 | 37.3 ± 0.3 | 26.5 ± 0.3 | 41.0 ± 0.3 | 30.2 ± 0.3 | 20.1 ± 0.3 |
| **IX** | 53.5 ± 0.3 | 41.0 ± 0.3 | 30.5 ± 0.3 | 44.3 ± 0.3 | 36.3 ± 0.3 | 25.9 ± 0.3 |
| **X** | 48.0 ± 0.3 | 33.2 ± 0.3 | 24.6 ± 0.2 | 33.7 ± 0.3 | 25.1 ± 0.2 | 18.7 ± 0.2 |
| **XI** | 58.7 ± 0.3 | 45.5 ± 0.3 | 32.3 ± 0.3 | 46.1 ± 0.3 | 35.2 ± 0.3 | 22.0 ± 0.2 |
| **XII** | 49.5 ± 0.2 | 38.2 ± 0.2 | 26.1 ± 0.2 | 39.5 ± 0.2 | 29.0 ± 0.2 | 19.4 ± 0.2 |
| **XIII** | 50.0 ± 0.2 | 40.9 ± 0.2 | 29.1 ± 0.2 | 40.4 ± 0.2 | 32.0 ± 0.2 | 22.9 ± 0.2 |
| **XIV** | 58.5 ± 0.2 | 44.1 ± 0.2 | 29.2 ± 0.2 | 48.3 ± 0.3 | 35.5 ± 0.2 | 23.8 ± 0.2 |
| **XV** | 51.3 ± 0.2 | 40.5 ± 0.2 | 29.8 ± 0.2 | 43.5 ± 0.2 | 31.6 ± 0.2 | 22.4 ± 0.2 |
| **XVI** | 55.0 ± 0.2 | 41.5 ± 0.2 | 28.5 ± 0.2 | 43.0 ± 0.2 | 29.8 ± 0.2 | 20.3 ± 0.2 |
| **IV fr.** | 54.5 ± 0.9 | 33.3 ± 0.8 | 18.2 ± 0.7 | 39.4 ± 0.9 | 27.3 ± 0.8 | 21.2 ± 0.7 |
| **XII fr.** | 47.0 ± 0.6 | 37.9 ± 0.6 | 22.7 ± 0.5 | 39.4 ± 0.6 | 33.3 ± 0.6 | 21.2 ± 0.5 |

a. Numbering of 16 major yeast chromosomes by Roman numerals.

b. MD [7] and IUPred [8] are the two methods used to predict disorder; they tend to capture different “flavours” of protein disorder.

c. Fraction of proteins predicted to contain at least one long region of predicted disorder. We show three thresholds with respect to “long”: ≥30, ≥50, and ≥80 consecutive disordered residues.

### Table S3: Overrepresented GO terms for molecular function analysis of chromosome III.

| **GO ^a^** | **Description ^b^** | **p-value ^c^** | **Corr. p-value^d^** | **X ^e^** | **N ^f^** | **cluster freq.^g^** | **total freq. ^h^** | **Genes in test set ^i^** |
| --- | --- | --- | --- | --- | --- | --- | --- | --- |
|  |  |  |  |  |  |  |  |  |
| 15849 | organic acid transport | 7.37E-05 | 4.22E-02 | 8 | 85 | 7.77 | 1.37 | ADY2, PAT1,VBA3, ATG22, AGP1, ERS1, PHO87, PMP1 |
| 46942 | carboxylic acid transport | 1.58E-04 | 4.22E-02 | 7 | 71 | 6.80 | 1.14 | ADY2, PAT1, VBA3, ATG22, AGP1, ERS1, PHO87 |
| 48610 | reproductive cellular process | 1.92E-04 | 4.22E-02 | 12 | 213 | 11.65 | 3.43 | RIM1, FIG2, MSH3, STE50, HMRA1, KAR4, FUS1, FYV5, RRT12, BIK1, SPS22, CDC10 |
| 19236 | response to pheromone | 2.02E-04 | 4.22E-02 | 8 | 98 | 7.77 | 1.58 | FIG2, KIN82, STE50, NOT1, KAR4, FUS1, FYV5, CDC10 |
| 22414 | reproductive process | 3.34E-04 | 4.68E-02 | 13 | 260 | 12.62 | 4.19 | RIM1, STE50, MSH3, RRT12, BIK1, CDC10, FIG2, HMRA2, HMRA1, KAR4, FYV5,  FUS1, SPS22 |
| 51704 | multi-organism process | 3.94E-04 | 4.68E-02 | 9 | 136 | 8.74 | 2.19 | FIG2, STE50, NOT1, KAR4, FUS1, FYV5, BIK1, MAK32, CDC10 |
| 19953 | sexual reproduction | 4.81E-04 | 4.68E-02 | 13 | 270 | 12.62 | 4.35 | RIM1, NOT1, STE50, RRT12, BIK1, CDC10, KCC4, FIG2, KAR4, FYV5, FUS1, SPS22, BUD5 |
| 15711 | organic anion transport | 4.90E-04 | 4.68E-02 | 3 | 10 | 2.91 | 0.16 | ADY2, GIT1, PHO87 |
| 747 | conjugation with cellular fusion | 5.03E-04 | 4.68E-02 | 8 | 112 | 7.77 | 1.81 | FIG2, STE50, NOT1, KAR4, FUS1, FYV5, BIK1, CDC10 |
| 3 | reproduction | 4.93E-02 | 1.70E+01 | 17 | 427 | 16.50 | 6.88 | RIM1, NOT1, STE50, MSH3, RRT12, BIK1, CDC10, KCC4, FIG2, HMRA2, HMRA1, KA4, FUS1, FYV5, SPS22, BUD3, BUD5 |

a. GO is a gene ontology number. Reproduction activity is most represented with 7 terms (48610, 19236, 22414, 19953, 747, 3 and 51704).

b. Description is a term describing a GO number.

c. p-value is assigned by Bingo[9], which is a tool to assess GO terms that are statistically significantly overrepresented in a set of genes. For example, organic acid transport is significantly overrepresented (15849).

d. Corr. p-value is a p-value after selected correction (FDR) that eliminates false positives. After the correction all terms have a similar p-value, around 4E-02.

e. X is the number of genes in a corresponding cluster (set of genes of Chromosome III) annotated to a certain GO term. The most represented GO term is reproduction (GO:000003; 17 genes). The total number of genes from Chromosome III considered for this clustering was 103.

f. N marks the number of genes in the reference set (6202) annotated to a certain GO term and selected automatically by Bingo. Reproduction is a GO term assigned to the largest number of proteins.

g. Cluster freq. marks the percentage of genes belonging to a particular GO term over the total number of genes considered for this clustering (103). The percentage is independent of the size of the cluster.

h. Total freq. marks the percentage of genes (N) in the reference set assigned to a certain GO term over the total number of genes in the reference set (6202).

i. Genes in test set mark the genes assigned to a specific GO term.

### Table S4: Overrepresented GO terms for biological process analysis of chromosome III.

| **GO ^a^** | **Description ^b^** | **p-value ^c^** | **Corr. p-value ^d^** | **X ^e^** | **N ^f^** | **cluster freq.^g^** | **total freq.^h^** | **Genes in test set ^i^** |
| --- | --- | --- | --- | --- | --- | --- | --- | --- |
|  |  |  |  |  |  |  |  |  |
| 18545 | NAD(P)H nitroreductase activity | 2.54E-04 | 2.03E-02 | 2 | 2 | 2.04 | 0.03 | HBN1, FRM2 |
| 8514 | organic anion transmembrane transporter activity | 2.54E-04 | 2.03E-02 | 2 | 2 | 2.04 | 0.03 | ADY2, GIT1 |
| 16657 | oxidoreductase activity, acting on NADH or NADPH, nitrogenous group as acceptor | 2.54E-04 | 2.03E-02 | 2 | 2 | 2.04 | 0.03 | HBN1, FRM2 |
| 16435 | rRNA (guanine) methyltransferase activity | 7.55E-04 | 3.62E-02 | 2 | 3 | 2.04 | 0.05 | BUD23, SPB1 |
| 9922 | fatty acid elongase activity | 7.55E-04 | 3.62E-02 | 2 | 3 | 2.04 | 0.05 | ELO2, APA1 |

a. GO is a gene ontology number.

b. Description is a term describing a GO number.

c. p-value is assigned by Bingo[9], which is a tool to assess GO terms that are statistically significantly overrepresented in a set of genes. For example, the rRNA (guanine) methyltransferase (16435) and fatty acid elongase activity (9922).

d. Corr. p-value, marked the p-value after using the selected correction (FDR) to eliminate false positives. After the correction all the terms have a similar p-value around 2-3 E-02.

e. X assigned the number of genes in this cluster (set of genes of Chromosome 3) annotated to a certain GO class. In this case all the GO class have the same number of elements. The total of genes from Chromosome III considered for this clustering was 98.

f. N marks the number of genes in the reference set (6116) annotated to a certain GO class and are selected automatically by bingo. In this case all the GO terms contain equal number of elements. For the GO terms 18545, 8514, 16657 all the possible genes in the reference set that are elements of these terms are in chromosome III.

g. Cluster freq. marks the percentage of genes belonging to a particular GO term over the total number of genes considered for this clustering (103). The percentage is independent of the size of the cluster.

h. Total freq. marks the percentage of genes (N) in the reference set assigned to a certain GO term over the total number of genes in the reference set (6116).

i. Genes in test set mark the genes assigned to a specific GO term.

### Table S5: Overrepresented GO terms for molecular function of the duplicated fragment of chromosome IV.

| **GO ^a^** | **Description ^b^** | **p-value ^c^** | **Corr. p-value^d^** | **X ^e^** | **N ^f^** | **cluster freq.^g^** | **total freq. ^h^** | **Genes in test set ^i^** |
| --- | --- | --- | --- | --- | --- | --- | --- | --- |
|  |  |  |  |  |  |  |  |  |
| 15749 | monosaccharide transport | 4.72E-05 | 7.13E-03 | 3 | 24 | 15.79 | 0.39 | HXT3, HXT7,  HXT6 |
| 8645 | hexose transport | 4.72E-05 | 7.13E-03 | 3 | 24 | 15.79 | 0.39 | HXT3, HXT7,  HXT6 |
| 8643 | carbohydrate transport | 2.41E-04 | 2.42E-02 | 3 | 41 | 15.79 | 0.66 | HXT3, HXT7,  HXT6 |

a. GO is a gene ontology number.

b. Description is a term describing a GO number.

c. p-value is assigned by Bingo[9], which is a tool to assess GO terms that are statistically significantly overrepresented in a set of genes. For example, the monosaccharide and hexose transport (15749, 8645).

d. Corr. p-value, marked the p-value after using the selected correction (FDR) to eliminate false positives. After the correction the GO terms 15749 and 8645 have the higher p-value 7.13E-03.

e. X assigned the number of genes in this cluster (set of genes of fragment of Chromosome IV) annotated to a certain GO class. The total of genes from Chromosome IV considered for this clustering was 19.

f. N marks the number of genes in the reference set (6208) annotated to a certain GO class and are selected automatically by bingo. In this case the carbohydrate transport GO class contains the largest number of elements.

g. Cluster freq. marks the percentage of genes belonging to a particular GO term over the total number of genes considered for this clustering (19). The percentage is independent of the size of the cluster..

h. Total freq. marks the percentage of genes (N) in the reference set assigned to a certain GO term over the total number of genes in the reference set (6209).

i. Genes in test set mark the genes assigned to a specific GO term.

### Table S6: Overrepresented GO terms for biological process of the duplicated fragment of chromosome IV.

| **GO ^a^** | **Description ^b^** | **p-value ^c^** | **Corr. p-value^d^** | **X ^e^** | **N ^f^** | **cluster freq.^g^** | **total freq. ^h^** | **Genes in test set ^i^** |
| --- | --- | --- | --- | --- | --- | --- | --- | --- |
|  |  |  |  |  |  |  |  |  |
| 5355 | glucose transmembrane transporter activity | 1.38E-05 | 2.61E-04 | 3 | 16 | 15.79 | 0.26 | HXT3, HXT7, HXT6 |
| 5353 | fructose transmembrane transporter activity | 1.38E-05 | 2.61E-04 | 3 | 16 | 15.79 | 0.26 | HXT3, HXT7, HXT6 |
| 15578 | mannose transmembrane transporter activity | 1.38E-05 | 2.61E-04 | 3 | 16 | 15.79 | 0.26 | HXT3, HXT7, HXT6 |
| 15149 | hexose transmembrane transporter activity | 2.01E-05 | 2.61E-04 | 3 | 18 | 15.79 | 0.29 | HXT3, HXT7, HXT6 |
| 15145 | monosaccharide transmembrane transporter activity | 2.01E-05 | 2.61E-04 | 3 | 18 | 15.79 | 0.29 | HXT3, HXT7, HXT6 |
| 51119 | sugar transmembrane transporter activity | 5.58E-05 | 6.05E-04 | 3 | 25 | 15.79 | 0.41 | HXT3, HXT7, HXT6 |
| 8262 | importin-alpha export receptor activity | 1.38E-05 | 2.61E-04 | 1 | 2 | \| 5.26 \| \| --- \| | 0.26 | MSN5 |
| 5049 | nuclear export signal receptor activity | 1.38E-05 | 2.61E-04 | 1 | 2 | 5.26 | 0.26 | MSN5 |

a. GO is a gene ontology number, the sugar transmembrane transport activity is the most represented with 6 terms (5355, 5353, 15578, 15149, 15145, 51119).

b. Description is a term describing a GO number.

c. p-value is assigned by Bingo[9], which is a tool to assess GO terms that are statistically significantly overrepresented in a set of genes. For example, the sugar transmembrane transporter activity (51119) is the most overexpressed term (6 E-05), this term is placed on the top of the hierarchy of the considered overrepresented GO terms for sugar transmembrane transport.

d. Corr. p-value, marked the p-value after using the selected correction (FDR) to eliminate false positives. After the correction all the terms have a similar p-value around 2-6 E-04.

e. X assigned the number of genes in this cluster (set of genes of fragment of Chromosome IV) annotated to a certain GO class. The most represented GO terms are the related to sugar transport activity; all included the same genes (HXT3, HXT7 and HXT6). The total of genes from Chromosome III considered for this clustering was 19.

f. N marks the number of genes in the reference set (6122) annotated to a certain GO class and are selected automatically by bingo. In this case the sugar transmembrane transporter activity GO class contains the largest number of elements (25 genes) and is placed on the top of the hierarchy of the considered overrepresented GO terms for sugar transmembrane transport.

g. Cluster freq. marks the percentage of genes belonging to a particular GO term over the total number of genes considered for this clustering (19). The percentage is independent of the size of the cluster.

h. Total freq. marks the percentage of genes (N) in the reference set assigned to a certain GO term over the total number of genes in the reference set (6122)

i. Genes in test set mark the genes assigned to a specific GO term.

### Table S7: Overrepresented GO terms for biological process of the duplicated fragment of chromosome XII.

| **GO ^a^** | **Description ^b^** | **p-value ^c^** | **Corr. p-value^d^** | **X ^e^** | **N ^f^** | **cluster freq.^g^** | **total freq. ^h^** | **Genes in test set ^i^** |
| --- | --- | --- | --- | --- | --- | --- | --- | --- |
|  |  |  |  |  |  |  |  |  |
| 17150 | tRNA dihydrouridine synthase activity | 6.53E-04 | 4.76E-02 | 2 | 4 | 4.76 | 0.07 | DUS3, DUS4 |

a. GO id assigned by gene ontology, only one activity is considered as overrepresented the tRNA dihydrouridine synthase activity (17150).

b. Description is a term describing a GO number.

c. p-value is assigned by Bingo[9], which is a tool to assess GO terms that are statistically significantly overrepresented in a set of genes. In this case, around 7E-04

d. Corr. p-value, marked the p-value after using the selected correction (FDR) to eliminate false positives. After the correction the p-value is around 5 E-02.

e. X assigned the number of genes in this cluster (set of genes of fragment of Chromosome XII) annotated to a certain GO class. The total of genes from fragment of Chromosome XII considered for this clustering was 42. It was a large number if compared with the fragment of chromosome IV (19 genes) but only one activity with only two genes was considered as overrepresented.

f. N marks the number of genes in the reference set (6122) annotated to a certain GO class and are selected automatically by bingo.

g. Cluster freq. marks the percentage of genes belonging to a particular GO term over the total number of genes considered for this clustering (42). The percentage is independent of the size of the cluster.

h. Total freq. marks the percentage of genes (N) in the reference set assigned to a certain GO term over the total number of genes in the reference set (6122)

i. Genes in test set mark the genes assigned to a specific GO term

### Table S8: Heat shock proteins distribution on chromosomes.

| **Id ^a^** | **chrom. ^b^** | **seq. start ^c^** | **seq. end ^d^** | **expr.levels ^e^** |
| --- | --- | --- | --- | --- |
| HSP71 | 1 | 139503 | 141431 |  |
| HSP73 | 2 | 84499 | 86448 |  |
| HSP26 | 2 | 382030 | 382674 | overexpr. |
| FES1 | 2 | 443821 | 444693 |  |
| CNS1 | 2 | 549771 | 550928 |  |
| HSP79 | 2 | 573915 | 575996 |  |
| HSP30 | 3 | 156109 | 157107 | **underexpr.** |
| TAH1 | 3 | 224399 | 224734 |  |
| HSP75 | 4 | 44065 | 45906 |  |
| MRH1 | 4 | 508147 | 509109 |  |
| CDC37 | 4 | 790328 | 791848 |  |
| HSP42 | 4 | 806621 | 807748 | overexpr. |
| AHA1 | 4 | 892875 | 893927 |  |
| HSP78 | 4 | 971808 | 974243 | **underexpr.** |
| HSP31 | 4 | 1501447 | 1502160 |  |
| HSP7E | 5 | 94644 | 96578 |  |
| HSP74 | 5 | 364589 | 366517 |  |
| HSP12 | 6 | 107256 | 107585 | overexpr. |
| HSP36 | 7 | 882812 | 883810 | overexpr. |
| HSP48 | 7 | 883810 | 882812 | overexpr. |
| PIH1 | 8 | 176965 | 177999 |  |
| SNL1 | 9 | 321454 | 321933 |  |
| SSC1 | 10 | 519638 | 521602 |  |
| HSP150 | 10 | 120449 | 121690 | **underexpr.** |
| HSP104 | 12 | 88623 | 91349 | overexpr. |
| HSP72 | 12 | 95566 | 97485 |  |
| HSP60 | 12 | 663284 | 665002 | overexpr. |
| HSC82 | 13 | 632355 | 634472 | overexpr. |
| HCH1 | 14 | 108467 | 108928 |  |
| HSP40 | 14 | 507097 | 505868 | overexpr. |
| HSP10 | 15 | 370524 | 370844 | overexpr. |
| STI1 | 15 | 381053 | 382822 |  |
| HSP33 | 15 | 1078545 | 1079258 |  |
| HSP32 | 16 | 11887 | 12600 |  |
| HSP82 | 16 | 96496 | 98625 | overexpr. |
| HSP7F | 16 | 350194 | 352275 |  |

a. Id is the standard gene name. Rows in grey are the verified HSPs in the *Saccharomyces* genome database (http:// yeastgenome.org).

b. Chrom. marks the chromosome that codified a certain protein.

c. Seq. start marks the first position of a region that codifies a certain protein on the chromosome.

d. Seq. end. marks the last position of a region that codifies for a certain protein on the chromosome.

e. Expr. levels mark the verified HSP proteins (grey) that are overexpressed after the trisomy is replaced by a refined local and less expensive solution. While the trisomy was used for survival to the heat-shock stress, these proteins were normally expressed ([10]). Curiously, HSP30 was the only gene localized on the duplicated chromosome III that was underexpressed after the trisomy was eliminated. The codified protein is localized in membrane and has several activities related to the ATPase activity and entry of the cell into stationary phase (response to starvation by arresting growth). The same expression level behavior is observed for HSP150 (chromosome X), which is a protein required for cell wall stability, and HSP78 (chromosome IV), which is a protein preventing the aggregation of misfolded proteins, as well as clear protein aggregates in mitochondria.

# References for Supplemetary Material

1. Tarca AL, Bhatti G, Romero R. A comparison of gene set analysis methods in terms of sensitivity, prioritization and specificity. PloS one. 2013;8(11):e79217. Epub 2013/11/22. doi: 10.1371/journal.pone.0079217

PONE-D-13-31980 [pii]. PubMed PMID: 24260172; PubMed Central PMCID: PMC3829842.

2. Panaretou B, Piper PW. The plasma membrane of yeast acquires a novel heat-shock protein (hsp30) and displays a decline in proton-pumping ATPase levels in response to both heat shock and the entry to stationary phase. Eur J Biochem. 1992;206(3):635-40. Epub 1992/06/15. PubMed PMID: 1535043.

3. Piper PW, Ortiz-Calderon C, Holyoak C, Coote P, Cole M. Hsp30, the integral plasma membrane heat shock protein of Saccharomyces cerevisiae, is a stress-inducible regulator of plasma membrane H(+)-ATPase. Cell Stress Chaperones. 1997;2(1):12-24. Epub 1997/03/01. PubMed PMID: 9250391; PubMed Central PMCID: PMC312976.

4. Piper PW, Talreja K, Panaretou B, Moradas-Ferreira P, Byrne K, Praekelt UM, et al. Induction of major heat-shock proteins of Saccharomyces cerevisiae, including plasma membrane Hsp30, by ethanol levels above a critical threshold. Microbiology. 1994;140 ( Pt 11):3031-8. Epub 1994/11/01. PubMed PMID: 7812443.

5. Seymour IJ, Piper PW. Stress induction of HSP30, the plasma membrane heat shock protein gene of Saccharomyces cerevisiae, appears not to use known stress-regulated transcription factors. Microbiology. 1999;145 ( Pt 1):231-9. Epub 1999/04/17. PubMed PMID: 10206703.

6. Goldberg T. HM, Hamp T., Karl T., Yachdav G., Ahmed N., Altermann U., Angerer P., Ansorge S., Balasz K. LocTree3 prediction of localization. Nucleic Acids Res. 2014. doi: 10.1093/nar/gku396.

7. Schlessinger A, Punta M, Yachdav G, Kajan L, Rost B. Improved disorder prediction by combination of orthogonal approaches. PloS one. 2009;4(2):e4433. Epub 2009/02/12. doi: 10.1371/journal.pone.0004433. PubMed PMID: 19209228; PubMed Central PMCID: PMC2635965.

8. Dosztanyi Z, Csizmok V, Tompa P, Simon I. IUPred: web server for the prediction of intrinsically unstructured regions of proteins based on estimated energy content. Bioinformatics. 2005;21(16):3433-4. Epub 2005/06/16. doi: bti541 [pii]

10.1093/bioinformatics/bti541. PubMed PMID: 15955779.

9. Maere S, Heymans K, Kuiper M. BiNGO: a Cytoscape plugin to assess overrepresentation of gene ontology categories in biological networks. Bioinformatics. 2005;21(16):3448-9. Epub 2005/06/24. doi: bti551 [pii]

10.1093/bioinformatics/bti551. PubMed PMID: 15972284.

10. Yona AH, Manor YS, Herbst RH, Romano GH, Mitchell A, Kupiec M, et al. Chromosomal duplication is a transient evolutionary solution to stress. Proc Natl Acad Sci U S A. 2012;109(51):21010-5. Epub 2012/12/01. doi: 1211150109 [pii]

10.1073/pnas.1211150109. PubMed PMID: 23197825; PubMed Central PMCID: PMC3529009.
