# Supplementary material for: Protein disorder reduced in Saccharomyces cerevisiae to survive heat shock
Source: F1000Res. 2015 Nov 6;4:1222. [Version 1] doi: 10.12688/f1000research.7178.1 (PMC4670006; doi:10.12688/f1000research.7178.1)
Supplement: Peer review history

# Peer review: Referee response for version 1

**DOI:** 10.5256/f1000research.7734.r11125

**Published:** 2015-11-20

**Authors:** Anuj Kumar

**Affiliations:** 1 Department of Molecular, Cellular & Developmental Biology, University of Michigan, Ann Arbor, MI, USA

The manuscript by Vicedo and colleagues presents an interesting observation: the authors have examined chromosomal regions (all of chromosome III and fragments of chromosomes IV and XII) that are duplicated in *Saccharomyces cerevisiae* in response to sudden exposure to high temperature and find that these chromosomal sequences are significantly decreased for genes encoding proteins with long disordered regions. The authors further analyzed these duplicated regions and the encompassed genes for any enrichment in annotated GO terms, as well as for encoded protein positioning in interaction networks. The results do not indicate significant GO term enrichment and reveal that the encoded proteins exhibit a decreased number of interactions per protein. The biological advantage to this duplication remains unclear.

Comments/suggestions:

The main conclusion presented here is interesting, but as the authors themselves attest, this observation does not explain a biological advantage behind the duplication.

On p. 4, the authors state that introducing an extra copy of *HSP30* into wild-type yeast does not modify the ability of the cells to cope with high temperature. The inclusion of laboratory data considering the effect of adding an extra copy of genes or chromosomal regions corresponding to some of the duplicated sequences would strengthen the paper significantly. This seems to be the easiest way to address a biological effect from duplication of a given gene.

In regards to the analysis, are the observed GO function annotations enriched with respect to other chromosomes/segments as opposed to being enriched against the genome as a whole? If the advantage to the cell centered on the functions associated with the genes in the duplicated regions, then these regions relative to other regions may be enriched for a function. If I’m thinking of this correctly, that would be slightly different than comparing a region for enrichment against the whole genome. Maybe the authors could compare enrichment in one chromosome versus another or utilize a sliding window corresponding to the size of a duplicated fragment to identify regions that would be most enriched for some potentially interesting functions. That might be a more sensitive means of identifying a functional enrichment for the duplicated regions.

Typos/stylistic suggestions:

 

- on p.2, first line under Introduction: I think it would be sufficient to state “The baker’s yeast *Saccharomyces cerevisiae*” rather than the text in parentheses.
- on p. 3, fourth paragraph under “Duplications in response to high temperature reduce protein disorder”: the first sentence in this paragraph (“Assume a certain amount …”) needs to be reworded.
- on p. 4, first paragraph, line 16: delete “the” from “insignificant the finding”

I have read this submission. I believe that I have an appropriate level of expertise to confirm that it is of an acceptable scientific standard.

# Peer review: Referee response for version 1

**DOI:** 10.5256/f1000research.7734.r11121

**Published:** 2015-11-17

**Authors:** Paul Pavlidis

**Affiliations:** 1 Centre for High-Throughput Biology and Department of Psychiatry, University of British Columbia, Vancouver, BC, Canada

Vicedo *et al*. report a computational analysis sparked by the interesting findings of Yona *et al.* (2012) of yeast duplication of chrIII having a selective advantage in the face of heat stress. Yona *et al*. did not fully mechanistically explain the reason chrIII aneuploidy is the one selected for, so Vicedo *et al*. have proposed a hypothesis: that chrIII has a substantially lower number of disordered proteins. They test this computationally, followed by some additional bioinformatics and “by hand” characterization of chrIII genes (and some other regions of interest following from Yona *et al*.).

The difficulty here is assigning cause vs. “permissive”. As Vicedo *et al*. report, the disorder hypothesis has limited predictive value because chrX genes also have a low disorder (on average), so the size of the chromosome is posed as the other important variable. However, Vicedo *et al*. seem to be proposing that “low disorder” is good for heat resistance per se (I grant them this) – and that overexpression of low disorder proteins is even better. I have difficulty with this second step, because the way the experiment of Yona *et al*. was done, it could easily be that there are “heat resistance proteins” on chrIII and that the overall duplication of chrIII is tolerated in the context of the advantage of overexpression those genes. But if this was the end of the story it would be hard to make a determination of whether this is a viable hypothesis.

However, there is an obvious problem: the work of Yona *et al. *identified 17 genes on chrIII that appear to be the main culprits for the heat resistance (at least most of them). I see no mention of these 17 in Vicedo *et al*. nor of the 22 control genes tested by Yona *et al*. If Vicedo *et al. *are right then there should be a difference in the disorder of these two sets of proteins. Otherwise, the observations might still be relevant, but that the orderedness of chrIII proteins might be permissive for overexpression of the actual heat-resistance genes via aneuploidy. In that case it might be the rest of the proteins on chrIII that have the orderedness properties, not the 17. (Note that I was not familiar with the Yona work before this review and I have not checked to see if Yona *et al. *or others have done any followup.)

Given the omission of discussion of the 17, the sections of this paper on network analysis, GO and localization cannot be interpreted with confidence. While I have some quibbles about them I would rather wait to see the response to the comments above.

I have read this submission. I believe that I have an appropriate level of expertise to confirm that it is of an acceptable scientific standard, however I have significant reservations, as outlined above.

# Peer review: Referee response for version 1

**DOI:** 10.5256/f1000research.7734.r11122

**Published:** 2015-11-17

**Authors:** Melchor Sanchez-Martinez

**Affiliations:** 1 Mind the Byte, Barcelona, Spain

The research article entitled 'Protein disorder reduced in *Saccharomyces cerevisiae *to survive heat shock' by Vicedo, Rost and co-workers shows how *Saccharomyces cerevisiae *reduces protein disorder to survive heat shock. It constitutes an interesting example about the usage of bioinformatic techniques to analyze protein disorder and its implications at a whole proteome level. In the article, there is a comprehensive explanation of study design, methods and analysis. The conclusions are well explained and justified on the basis of the results.

Consequently, the manuscript is recommended for approval. It is a good piece of science that meets the indexation requirements of F1000Research.

However I have some comments that the authors may consider and/or answer.

1. As far as I know except some rare exceptions the protein disorder is reduced as temperature increases, oppositely as happens with ordered proteins or protein regions. With increasing temperature, disordered proteins and regions tend to adopt a transitory structure. Commonly this transitory structure is necessary for proteins to perform its biological function. In other recent works that the authors have published have published (Reference 37 in the References section), they stated that "protein disorder appeared as a possible building block to bring about evolutionary changes such as the adaptation to different habitats" and in that sense seems that more disorder should imply a better response to heat shock.
  Thus is surprising for me that in response to heat there is a protein disorder reduction, whereas I expect a disorder increment. Why does it happens? Maybe the answer is so easy as that the disordered proteins do not help to "fight" against heat shock or as the authors said "...Some of these proteins with long disordered regions might not work correctly in heat...", but  I am curious about that. Do you have any evidence or supported hypothesis to explain that?
2. Regarding to authors statement "...Some of these proteins with long disordered regions might not work correctly in heat...", a plausible way to study that and obtain a more conclusive answer could be to perform a molecular simulation. Maybe a Replica Exchange Molecular Dynamics or Monte Carlo simulations could give a better understanding of what happens with these protein at high temperatures.

I have read this submission. I believe that I have an appropriate level of expertise to confirm that it is of an acceptable scientific standard.
